# Supplementary material for: Point-of-care diagnostic technology in paediatric ambulatory care: a qualitative interview study of English clinicians and stakeholders
Source: BMJ Open. 2022 Jun 7;12(6):e059103. doi: 10.1136/bmjopen-2021-059103 (PMC9174781; doi:10.1136/bmjopen-2021-059103)
Supplement: Supplementary data [file bmjopen-2021-059103supp001.pdf]

Supplementary Materials: Interview Topic Guide

Version 1.1 09.01.2020

Thank you for participating in our study. Any questions about the information booklet?

Background
Our aim is to understand when, where, how and why point-of-care tests and technologies in ambulatory paediatrics could be useful. By ambulatory settings, we mean primary care, emergency departments and out-of-hours services. By point-of-care tests, we mean any diagnostic technology to include tests on bodily fluids, imaging, wearables, digital technology, and smart phone apps.

Consent form

Interview
Recording now….

All:

- What is your job description?
- How many years' experience do you have in that role?
- Age
- What area of the country do you work in?
- Would you say that your work setting is:
  Rural Urban
  Primary Care Secondary Care Tertiary Care
  (Stakeholder)

All:
What do you think about bedside **TESTS** in children in ambulatory care settings?

What experience do you have of these?

What current point-of-care tests are currently useful in clinical practice (stakeholders: have you heard of existing tests being useful)?
Probe e.g. urine dip, blood glucose check

How have these tests been helpful in your clinical practice (stakeholders: have you heard of existing tests being useful)? Please give an example. How did it change what you did?

Do you have tests available to you that aren't useful (stakeholders: are there tests that aren't useful)? Please give an example.
Probe: BM when not confident to do a finger prick, too time consuming
Reasons why not

Have **TECHNOLOGIES** ever been helpful in your clinical practice? Example if yes.
Probe: apps for fitting, temperature monitors

Clinicians:

What training have you had in the use of POC testing/technology?

Which clinical pathways might benefit from a new test/technology?

Can you think of a recent specific situation in which it would have been useful to have a novel point-of-care test or technology?

How would that be useful? How would it change what you would do, or the patient outcome?

Probe:

for:

- *Decision making e.g.*
  - *Treatment given e.g. Antibiotic prescribing*
  - *Predicting severity of illness*
  - *Referral to secondary care*
  - *Admission to hospital*
  - *Self-management*
  - *Hospital management- investigations, treatments, referral, surgical, length of stay*
- *Waiting times*
- *Explanation of treatment plan*

Where would you see them fitting in the pathway of patient flow?

What would be key characteristics of a successful paediatric diagnostic?

Probe: "facilitators" in terms of….

- *time taken to perform the test*
- *time taken to obtain the result*
- *cost*
- *route of sampling*
- *amount of tests able to perform simultaneously (e.g. panel)*
- *Acceptability: Who would perform? (Ease of use), Novelty, trust*
- *Sensitivity and specificity (uncertainty)*

What impact might POC tests/technology have on *parents*? What would they need to know?

What *disadvantages* might POC tests/technology in paediatrics in ambulatory settings have?

Probe: barriers to use

Are you aware of any new promising diagnostics in development coming out in your work place?

Do you have any other thoughts or ideas?

Any questions for me?

Is there anyone else that you can think of who might want to contribute**?**

Admin

I will send Amazon voucher by email

Would you like a CPD certificate to acknowledge your contribution?
