# Supplementary material for: Point-of-care diagnostic technology in paediatric ambulatory care: a qualitative interview study of English clinicians and stakeholders
Source: BMJ Open. 2022 Jun 7;12(6):e059103. doi: 10.1136/bmjopen-2021-059103 (PMC9174781; doi:10.1136/bmjopen-2021-059103)
Supplement: Author's manuscript [file bmjopen-2021-059103.draft_revisions.pdf]

BMJ Open is committed to open peer review. As part of this commitment we make the peer review history of every article we publish publicly available.

When an article is published we post the peer reviewers' comments and the authors' responses online. We also post the versions of the paper that were used during peer review. These are the versions that the peer review comments apply to.

The versions of the paper that follow are the versions that were submitted during the peer review process. They are not the versions of record or the final published versions. They should not be cited or distributed as the published version of this manuscript.

BMJ Open is an open access journal and the full, final, typeset and author-corrected version of record of the manuscript is available on our site with no access controls, subscription charges or pay-per-view fees (http://bmjopen.bmj.com).

If you have any questions on BMJ Open's open peer review process please email info.bmjopen@bmj.com

Point-of-care Diagnostic Technology in Paediatric Ambulatory Care: a Qualitative Interview study of Clinicians and Stakeholders

Journal:	*BMJ Open*
Manuscript ID	bmjopen-2021-059103
Article Type:	Original research
Date Submitted by the Author:	18-Nov-2021
Complete List of Authors:	Raymond, Meriel; University of Oxford, Nuffield Department of Primary Care Health Sciences<br>Bird, Chris; Birmingham Children's Hospital, Emergency Department; University of Oxford Nuffield Department of Primary Care Health Sciences<br>van Hecke, Oliver; Oxford University, Nuffield Department of Primary Care Health Sciences<br>Glogowska, Margaret; Oxford University, Nuffield Department of Primary Care Health Sciences<br>Hayward, Gail; Oxford University, Nuffield Department of Primary Care Health Sciences
Keywords:	Molecular diagnostics < INFECTIOUS DISEASES, Paediatric A&E and ambulatory care < PAEDIATRICS, QUALITATIVE RESEARCH, PRIMARY CARE

SCHOLARONE™ Manuscripts

I, the Submitting Author has the right to grant and does grant on behalf of all authors of the Work (as defined in the below author licence), an exclusive licence and/or a non-exclusive licence for contributions from authors who are: i) UK Crown employees; ii) where BMJ has agreed a CC-BY licence shall apply, and/or iii) in accordance with the terms applicable for US Federal Government officers or employees acting as part of their official duties; on a worldwide, perpetual, irrevocable, royalty-free basis to BMJ Publishing Group Ltd ("BMJ") its licensees and where the relevant Journal is co-owned by BMJ to the co-owners of the Journal, to publish the Work in this journal and any other BMJ products and to exploit all rights, as set out in our licence.

The Submitting Author accepts and understands that any supply made under these terms is made by BMJ to the Submitting Author unless you are acting as an employee on behalf of your employer or a postgraduate student of an affiliated institution which is paying any applicable article publishing charge ("APC") for Open Access articles. Where the Submitting Author wishes to make the Work available on an Open Access basis (and intends to pay the relevant APC), the terms of reuse of such Open Access shall be governed by a Creative Commons licence – details of these licences and which Creative Commons licence will apply to this Work are set out in our licence referred to above.

Other than as permitted in any relevant BMJ Author's Self Archiving Policies, I confirm this Work has not been accepted for publication elsewhere, is not being considered for publication elsewhere and does not duplicate material already published. I confirm all authors consent to publication of this Work and authorise the granting of this licence.

For peer review only

Point-of-care Diagnostic Technology in Paediatric Ambulatory Care: a Qualitative Interview study of Clinicians and Stakeholders

Dr. Meriel Raymond*,
Dr. Chris Bird,
Dr. Oliver Van Hecke,
Dr. Margaret Glogowska,
Professor Gail Hayward

Nuffield Department of Primary Care Health Sciences,
University of Oxford, Oxford, UK

*Corresponding author. Tel: +44(0)1865-289300

E-mail: meriel.raymond@phc.ox.ac.uk orcid.org/0000-0002-0442-9280

Keywords
Bedside
Diagnostics
Paediatrics
Ambulatory care facilities
Qualitative

For peer review only

Abstract

Point-of-care (POC) tests have the potential to improve paediatric healthcare. However, both the development and evaluation of POC technology have almost solely been focussed on adults.

Objectives

We aimed to explore frontline clinicians' and stakeholders' current experience of POC diagnostic technology in children, and to identify areas of unmet need.

Design, setting and participants

Twenty-two qualitative semi-structured telephone interviews were carried out with purposively sampled participants from clinical paediatric ambulatory care and charity, industry and policymaking stakeholders. The interviews were audio-recorded and transcribed. The transcripts were analysed thematically.

Results

The main perceived benefits of POC tests and technologies were that they aided early decision-making and could be convenient and empowering when used independently by patients and their families. Clinicians and stakeholders wanted more POC tests to be available for use in clinical practice. Most recognised that play and reward are important components of successful POC tests for children. Clinicians wanted tests to give them answers which would result in a change in their clinical management. Detecting acute serious illness, notably distinguishing between viral and bacterial infection, was perceived to be an area where tests could add value. POC tests were thought to be particularly useful for children presenting atypically where diagnosis was more challenging, such as those less able to communicate, and for rare and serious diseases. Many participants felt they could be useful in managing chronic disease.

Conclusions

This exploratory study found that clinicians and stakeholders supported the use of diagnostic POC technology in paediatric ambulatory care settings in England. Some existing tests are not fit for purpose and could be refined. Industry should be encouraged to develop new child-friendly tests to tackle areas of unmet need, guided by the preferred characteristics of those working on the ground.

Funding

This study was funded by the NIHR and NIHR CH MIC.

For peer review only

Article Summary

Strengths and limitations of this study

- Semi-structured interviews enabled an in-depth exploration of the experiences of the heterogeneous participants with different backgrounds (1).
- Purposive sampling with snowballing facilitated the interviewing of a broad range of clinicians and stakeholders on this topic. Inclusion of stakeholders enabled emergence of views from policymaking and industry perspectives.
- The breadth of the study limited the depth to which we could explore any specific clinical presentations or contexts and made “data saturation” (2) difficult to achieve.
- Although children’s and parents’ perspectives were mentioned by our participants, and some offered their own experiences as parents; their views were not specifically sought in this study.
- All participants were based in England. As such our findings are applicable to English stakeholders and clinicians and may not be transferable to other settings.

Introduction

Paediatric ambulatory care places huge demand on healthcare services. One in four consultations in ambulatory care in the UK are for children (3,4). Children present with a different disease spectrum to adults, having a high incidence of acute infections (5). Most of these consultations are for upper respiratory tract infections which are generally self-limiting. The incidence of serious infection in children presenting to primary care has been estimated to be less than 1%. The challenge in primary care is that these serious infections often present with non-specific symptoms, especially in the early stages. Furthermore, children have the potential to deteriorate more quickly than adults (6). It is difficult to detect those children who will progress to serious illness requiring secondary care input (7) in a timely way. Inappropriate prescribing, unnecessary referrals to hospital and needless additional testing often result from this diagnostic uncertainty (8). There was a 10–20% trend increase in potentially avoidable, short stay hospital admissions of children from 1997–2012 (9–11). The onset the SARS-CoV-2 pandemic in March-April 2020 saw 69% less children attending emergency departments in the UK (12); this was followed by a 1-4% increase in attendance per week. Paediatric emergency research groups have identified the need to develop better diagnostics for “low numbers, high stakes diagnoses” in children (13–15).

POC (point-of-care) tests can be defined as any test where the results are available during a clinical visit, without needing to send a sample to a laboratory (16). They have the potential to reduce diagnostic uncertainty in acute illness and streamline management of chronic disease, improving clinical outcomes and reducing health-related costs. Yet, there have been very few studies, limited to a handful of diseases (mostly malaria and HIV in resource-limited settings), which have shown the potential of benefit of point of care tests in paediatric populations (7).

For peer review only - http://bmjopen.bmj.com/site/about/guidelines.xhtml

Attitudes of primary care clinicians towards POC blood tests in Europe and Australia have been synthesised in one systematic review of qualitative studies (16). Participants thought that POC testing improved diagnostic certainty, treatment, self-management of chronic disease, clinician-patient relationships, and perceived patient experience. The views of English paediatricians (17) and English Emergency Department healthcare providers (18) on the use of POC tests to assess febrile children have also been explored. Little is known about attitudes of primary care clinicians towards POC tests more broadly in children; or those of hospital clinicians towards POC tests for afebrile children. There is little information on stakeholders' views; or views towards POC technologies, including apps and wearables.

The diagnostic needs in paediatric ambulatory care are unlikely to be met by diagnostics which have been developed with an adult population primarily in mind. In order to stimulate the development and evaluation of POC diagnostic technology which is of greatest benefit in paediatric healthcare it is important to understand the current experience of those using these technologies and identify areas of unmet need. We aimed to seek the views and experiences of a broad range of clinicians and stakeholders with an interest in paediatric ambulatory care in the UK about current usage and unmet needs for POC diagnostic technology.

Methods

Qualitative research methodology was used in this study. Qualitative research is highly appropriate for capturing and exploring people's experiences and perceptions; and has considerable power to explain actions, decisions and processes (2). A qualitative methodology is therefore appropriate to explore perceptions of clinicians and stakeholders towards POC tests and technologies in paediatric ambulatory care.

Sampling and recruitment

A maximum variation, purposive sample of participants was sought based on gender, level of clinical experience, and range of NHS settings(19). We advertised for participants using the PERUKI (Paediatric Emergency Research in the UK and Ireland) mailing list in August 2019 and April 2020, and on the website for the Nuffield Department of Primary Care Health Sciences (NDPCHS), University of Oxford, from 19th June 2019 at www.phc.ox.ac.uk/iTAP.

We directly approached specialist clinicians, children's commissioners, CCGs (Clinical Commissioning Groups), children's charities pertaining to serious illness, and TITCH (Technology Innovation Transforming Child Health) using telephone or email details that were in the public domain.

Recruitment was extended to contacts of participants in a "snowballing" effect. Early interviews shaped the identification of further interviewees, using a principle of grounded theory; namely, theoretical sampling which permits the deliberate inclusion of participants whose viewpoints have been shown to be of interest(20).The decision to stop interviewing, when little new information was emerging and there was sufficient explanation for the emerging themes, was discussed and agreed among the research team.

Interviews

Qualitative semi-structured individual interviews were conducted by the primary researcher MR. Interviews were chosen in preference to questionnaires to enable in-depth exploration

For peer review only - http://bmjopen.bmj.com/site/about/guidelines.xhtml

of the experiences of the heterogeneous participants (1) , through interviewer and interviewee interaction, and exploration of details which were significant to either party as the interview progressed. A focus group discussion of a wide range of professionals would be less likely to capture these individual experiences. Focus-group discussion was also avoided due to logistical difficulty in arranging group clinician sessions; need for HRA (Health Research Authority) approval for interviews occurring on NHS (National Health Service) premises; and divergence of stakeholder interests.

Participants were offered a telephone or face-to-face interview that would take around 30 minutes. Informed verbal consent was obtained prior to interview.
Draft topic guides for the interviews with clinicians and stakeholders were developed to address the study objectives. The topic guide was initially reviewed by the research team; modified iteratively by the primary researcher based on feedback; and amended after 12 interviews following discussion with the research team.
Interviews were recorded using a digital audio-recorder and transcribed verbatim. Field notes were made by the primary researcher during and after the interviews. Data were stored and processed in line with GDPR (General Data Protection Regulation). In recognition of the time contributed to the study, interviewed participants were offered a £20 gift voucher.

Analysis

Transcripts were anonymised and checked against the audio recordings for accuracy. Anonymised transcripts were uploaded into a specialist software programme to assist organisation of data (NVivo version 12). A "ground up" approach from the data was adopted to analyse the complete data set (21) using thematic analysis (1). The primary researcher read and familiarised herself with the transcripts. Systematic and detailed codes were compared and grouped to create categories. These were organised into an initial "data driven" coding framework based on 6 coded interviews. These interviews were read by MG and GH and the coding framework checked. This coding framework was iteratively applied to subsequent transcripts. "Constant comparison" was used to cross-check ideas and categories that were emerging across interviews, taking an inductive approach (2). Broad themes were developed using "single sheet" brainstorming (2). Agreement on coding, themes, and subthemes, was sought between members of the research team. An audit trail from the raw data of the interview transcripts through coding to development of themes was established to ensure dependability. Participants provided feedback on the findings.

Researcher characteristics and reflexivity

The primary researcher was a General Practitioner undertaking a master's degree in public health. She attended a course on Qualitative Interviewing prior to the study. The participants were aware of her clinical background prior to interview and her reasons for undertaking the research.

Ethics

Ethical approval was obtained from the Medical Sciences Interdivisional Research Ethics Committee, University of Oxford, on the 30th April 2019 (reference R63109/RE001); and LSHTM (London School of Hygiene & Tropical Medicine) MSc Research Ethics Committee on the 14th May 2019 (reference 17436).

For peer review only - http://bmjopen.bmj.com/site/about/guidelines.xhtml

Public and patient involvement

No patients involved. The final manuscript was sent to participants.

Results

Twenty-two interviews were conducted between June 2019 and July 2020. Due to participant preference and the COVID-19 pandemic, all interviews were conducted by telephone.

Participant characteristics

For complete participant characteristics please see Table 1. Of the 22 participants, 14 were clinicians; three stakeholders; and five were both clinicians and stakeholders.
Of the 19 clinicians, nine were from primary care (seven GPs, two nurses), and ten from secondary or tertiary care (eight doctors, two nurses).
The eight stakeholders represented three CCGs (Clinical Commissioning Groups), three charities, and one tech company.

Themes and sub-themes

The main themes and sub-themes are described below in Table 2**Table 2** Main themes and subthemes .

Theme 1: Potential benefits of POC tests and technologies

1a: POC tests facilitate early decision-making

Participants reported that the predominant advantage of POC tests and technologies is that they give rapid results compared to tests requiring laboratory processing or transfer of the child to another department. They thought that POC tests increased the speed of clinicians' decisions and allowed the assessing clinicians to incorporate the result as part of their holistic assessment.

"you don't really know if this lump is an abscess or not, which can guide your treatment and management; having to rely on a radiologist really delays the treatment of the child and makes you… admit the child for the scan to happen the next day... …if you had the chance to do that by the bedside… that….would really make a difference [Emergency Department Consultant Clinician#6]

Delayed laboratory results would be more likely to be interpreted by a clinician who had not seen the child.

A Macmillan GP with palliative care as a specialist interest [Clinician#5] thought that availability of POC full blood count in primary care settings would facilitate faster pick-up of difficult-to-diagnose serious conditions such as childhood cancer, as a delay in hospital referral often delayed the diagnosis. Many clinicians and stakeholders thought that POC technologies could help to give earlier diagnosis of chronic disease, enabling prompt appropriate treatment and decreasing morbidity. Examples were given of spirometry and Fractional Exhaled Nitric Oxide (FeNO) [see Table 3]; POC eosinophils; and mental health questionnaires.

For peer review only - http://bmjopen.bmj.com/site/about/guidelines.xhtml

Clinicians and stakeholders representing children with additional needs, disabilities and life limiting conditions, added that early pick-up of clinical deterioration was particularly important, as they often had an *"up and down trajectory and a high risk of sudden episodes of acute illness"* [GP Clinician#5]. They thought it might be worth monitoring such children at home to pick up early physiological changes as a "*safety net*" [GP Clinician#5].

1b: Home-based POC tests are convenient

Participants suggested that POC tests performed at home by patients and their families or caregivers could decrease the need for face-to-face assessment in health care settings. An example was given of the use of POC clotting testing in children with replacement heart valves "*improv[ing] the quality of those families' lives*" making a "*really big difference*" [Community paediatrician Clinician#13]. Participants felt that home testing would be convenient for patients and clinicians and could speed up recognition and escalation of acute illness. Furthermore, it was thought that this would improve infection prevention and control, particularly during the COVID-19 pandemic. An unmet need was identified for the detection of vital signs including temperature and oxygen levels by parents at home, for example with smart-phone cameras (see Table 3).

1c: POC tests are empowering for children and their families

Participants explained that the additional objective information given by POC tests and technologies to children and their families would empower them to communicate their illness more effectively to health care professionals, facilitating the consultation. This was particularly important for the families or carers of children who struggled to communicate because of disability, and in whom detection of illness is more difficult.

"families find communication about a problem with healthcare services quite challenging and if they were equipped with a range of clinical parameters to help their discussion… they might find they access the right kind of healthcare quicker" [GP Clinician#5]

Furthermore, participants said that the results from these tests helped children with chronic disease and their families to look after their own health better.

"I have heard of young people using and parents taking control of diabetes management using Apps quite pro-actively…….[they attend] clinic and consultants [feel] a bit redundant because suddenly they've been replaced by this App which is giving their family a lot more control… [they] are actually making those decision themselves about management…we can… empower people to actually self-manage these conditions very effectively" [GP Clinician#5]

Theme 2: Areas for improvement for POC tests and technologies

2a: POC tests should be more widely available

Most of the participants had not come across many POC technologies in their clinical practice, or felt that were not widely available. They also thought that cost, for example of FeNO and peripheral oxygen saturation monitors, could limit accessibility and lead to "*inequitable distribution*" [Asthma nurse Clinician#4].

2b: End-users should find POC tests quick and easy to use

Many participants felt that POC tests and technologies need to be quick to use, so that a child could be distracted, for instance during a distressing test; or not lose concentration, for instance during measurement of peak flow. The "*time-poor*" clinicians [GP Clinician#9] also

For peer review only - http://bmjopen.bmj.com/site/about/guidelines.xhtml

wanted quick tests; firstly to improve patient flow, and secondly to enable continuity, in that the same clinician seeing the patient at initial contact could also be responsible for interpreting the result. Some participants expressed a preference for tests that would give results in seconds. Innovations they suggested included contactless scanning to measure oxygen saturations and height [Emergency Department nurse Clinician #15]; measurement of basic observations with smartphone cameras [GP Clinician #16] or use of smartphone apps to diagnose rashes [Advanced nurse practitioner Clinician#12].

Participants reported that samples need to be easy to obtain to avoid causing pain and stress for children and their families. This was particularly true for finger pricks, throat swabs and blood pressure measurements. There was however a consensus that finger prick tests using a single drop of blood are acceptable. Many participants stated that urine samples (see Table 3), peak flows and spirometry could be challenging for younger children to perform. Participants said that POC tests and technologies requiring no extra effort by the child would be ideal (see Table 3, smart inhaler and monitoring of exhaled gases).

Many participants felt that tests and technologies needed to be "*fool proof*" to perform [Emergency Department Consultant Clinician#6]. Participants reported that where tests were not easy to use, it put them off using them. They frequently gave the example of measuring peripheral oxygen saturations which posed a logistical challenge in primary care as it was often difficult to locate equipment and obtain a reliable result. One participant stated *"there's a gap of a non-single-use [oxygen saturation] probe that is effective and quick to use"* [Advanced nurse practitioner Clinician#17].

"With younger kids… under five years of age… and particularly babies under one… we've got one [Peripheral oxygen saturations monitor] machine per practice. So first of all, I have to go out and get it, find the box. It might be… in the right place or maybe another clinician's got it. You've got to send a message out, "Who's got the [oxygen saturations] machine?"… it seems to take… four or five minutes sometimes to get a reading. You fidget around, try on the thumb… end up trying earlobes and things… it's just really hard when, on young babies you try across the foot and the kid starts wriggling and kicking… and then if you're unlucky you'll get a bad trace and… it's not actually their sats because the pulse rate's completely wrong… but if it starts to then blip and say things like 80 per cent, you just start thinking, 'Oh God, why the hell did I do this" [GP Clinician#10]

2c: POC tests should be agreeable and engaging for children

Many participants felt that POC tests should ideally be enjoyable. The asthma nurse [Clinician#4] described making peak flows into a game. Reward was particularly important in children with disability.

"anything that could be done as a wearable, so that… they're still able to play. A lot of the kids that we have when they go into A&E, they might be really quite poorly but actually… it's usual for them… They just want to be able to play and… get on with their life…. ….and so it's then quite inconvenient and they get upset… and quite angry and quite stroppy… because… it's interfering with their day… anything that we can do to… make it less medicalised and more play-based, more fun [is] always a good thing". [Little Miracles Stakeholder#2]

For peer review only - http://bmjopen.bmj.com/site/about/guidelines.xhtml

Visual results such as FeNO were described as engaging the patient and increasing adherence with medication. When children entered information into one stakeholder's app, their progress was indicated by the growth of a plant [Stakeholder#4].

"FeNO is massively useful in patients that are… not adherent with their medication in that it gives them that lightbulb moment to actually visualise what's going on inside the chest… [if] you can then illustrate that by measuring an inflammatory marker, they tend to be a bit more adherent". [Asthma nurse Clinician#4]

2d: POC tests should make a difference to clinical management

Participants wanted POC tests and technologies to give them results that would make a difference to their decision-making and get them *"further ahead"* [Emergency Department Consultant Clinician #6]. They felt that "*something objective*" [GP Clinician#10] might "*stop interpersonal and intrapersonal variance"* [Paediatrician Clinician#2].Participants wanted confirmatory tests to enable detection of acute serious illness *"to rule out the worst-case scenario"* [Paediatric trainee Clinician#7]. For instance, many clinicians asserted that low peripheral oxygen saturations would help pick up acute serious illness, and guide referral to hospital, mode of transport to hospital, and need for admission. A GP [Clinician #9] had invested £500 in a machine because of this perceived impact. One participant [Paediatrician Clinician#2] felt that these basic observations were sometimes under-utilised in the clinical setting, and that this could be a focus for improvement over the development of new tests or technologies.

"I sometimes don't recognise that people are as bad as they are because I'm a bit too optimistic. But sometimes I'll see a child… and say, 'Actually, you don't look…too bad' and then I'll put the oximetry on and go… 'Oh, actually, you're worse than I realised. Let's just think about this a bit more seriously'" [GP Clinician #9]

The acute serious illnesses that participants raised were predominantly sepsis and meningitis, with an emphasis on the need to distinguish between bacterial and viral infection, and confirmation of a specific pathogen being particularly helpful. This could increase clinician confidence in diagnosis and management, including antibiotic prescribing. They gave examples of POC streptococcal PCR and POC respiratory PCR panels in primary care.

"URTI {Upper Respiratory Tract Infection}-type symptoms… the research nurse did [nasopharyngeal swabs] and they could run the analyser and within an hour you would know whether this had a bacterial element to it and then obviously you could prescribe [antibiotics] if that was appropriate… the parents [had] such a willingness to take part in that research trial… the fact that you could say to them, 'Yeah we can test you straight away now,' and we can get an answer to you… parents were very happy with that" [Advanced Nurse Practitioner Clinician#12]

The importance of exact pathogen detection in the context of public health was also raised, with implications for contact-tracing and vaccination when meningococci and SARS-CoV-2 were detected. Participants acknowledged that results might offer false reassurance, for example in a viral respiratory tract infection, and that clinicians would still need to safety net against development of a secondary bacterial infection. Desire for POC tests to assist in diagnosis of non-infective acute serious illness including ischaemia, diabetes, cancer, seizures, poisoning and trauma were also mentioned in the interviews; as were tests to

For peer review only - http://bmjopen.bmj.com/site/about/guidelines.xhtml

diagnose chronic disease such as asthma and genetic conditions. Suggestions for areas of innovation are listed with quotes in Table 4.

Discussion

Summary of main findings

There are areas of unmet need for POC tests in paediatric ambulatory care. Participants wanted more POC tests and technologies to be available. They thought they should be user-friendly and, where possible, fun. They felt that they could empower patients and their families when used at home; particularly in children with chronic disease. Clinicians wanted POC tests to give results that made a difference to clinical management; especially in the detection of acute serious illness in children for whom diagnosis is more challenging.

Comparison with existing literature

Many of our participants thought that POC tests could facilitate early clinical decision-making. This is in keeping with the findings from one qualitative systematic review assessing primary clinicians' attitudes towards POC blood tests in primary care settings in high income countries (16). In our study, participants placed new importance on the use of POC tests and technologies for earlier detection of acute serious illness in children who present atypically, and for whom diagnosis is normally delayed as a result. Our study highlighted that the convenient use of POC tests at home by patients and their families could bypass the need for clinician assessment and empower patients and families. This is in keeping with the NHS's promotion of Integrated Care Systems (22), and development of better diagnostics to improve diagnostic bottlenecks and help tackle health inequalities (23). Child health nurses have highlighted in an interview study that parents felt empowered by being able to take care of their child in a safe and structured way at home (24).

The preference of our participants for POC tests to be easy to use and avoid causing pain was also found in a study of English paediatricians (17). Their belief that finger prick testing is acceptable has similarly been demonstrated in GP settings(25). This study highlighted new information that play, visualisation and reward are important components of successful POC tests and technologies in children.

Implications of findings and recommendations

For industry

Participants wanted POC tests to be routinely available in clinical practice with the potential for tests to be used by children and their carers at home. For diagnostic developers, our study offers evidence in favour of the design of POC tests and technologies that incorporate play and reward to make them more acceptable to children and their carers.

For further research

Further qualitative research to evaluate preferred characteristics of POC tests and technologies from parents and children themselves is advised to guide future "patient-up" development by industry. This study highlighted that this would be particularly important in children who present atypically such as children with disability, and children diagnosed with cancer. This would enable more equitable representation of children with greater healthcare needs.

For peer review only - http://bmjopen.bmj.com/site/about/guidelines.xhtml

Word Count
3812 words

Acknowledgements
We acknowledge our participants who took the time to participate in our study.

Author contributions
MR- Interviews, Project administration, Data curation, Formal analysis, Writing – original draft, Writing – review & editing
CB- Writing – review & editing
OVH- Writing – review & editing
MG- Conceptualization, Methodology, Supervision, Writing – review & editing
GH- Conceptualization, Funding acquisition, Methodology, Supervision, Writing – review & editing

Data sharing statement
Participants gave their permission for the sharing of anonymised data. This will be subject to appropriate research requests, screened by a panel of the authorship team.

Funding
This research was funded by the National Institute for Health Research (NIHR) Community Healthcare MedTech and in Vitro Diagnostics Co-operative (CH MIC), award code MIC-2016-018. MR's salary was funded by an in-practice fellowship grant from the NIHR.

Transparency declarations
MR affirms that the manuscript is an honest, accurate and transparent account of the study being reported, that no important aspects of the study have been omitted, and that any discrepancies from the study as planned have been explained.

Disclaimer
The views expressed are those of the authors and not necessarily those of the NHS, the NIHR or the Department of Health and Social Care.

Competing interest
All authors have completed the ICMJE uniform disclosure form at http://www.icmje.org/disclosure-of-interest/ and declare: all authors had financial support from NIHR CH MIC for the submitted work; no financial relationships with any organisations that might have an interest in the submitted work in the previous three years; no other relationships or activities that could appear to have influenced the submitted work.

References
1. Flick U. The SAGE Handbook of Qualitative Data Analysis [Internet]. London; 2014. Available from: https://methods.sagepub.com/book/the-sage-handbook-of-qualitative-data-analysis
2. Silverman D. Doing qualitative research : a practical handbook . 3rd ed. London ; SAGE; 2010.
3. Hobbs FDR, Bankhead C, Mukhtar T, Stevens S, Perera-Salazar R, Holt T, et al. Clinical workload in UK primary care: a retrospective analysis of 100 million consultations in England, 2007-14. Lancet (London, England) [Internet]. 2016/04/05. 2016 Jun 4;387(10035):2323–30. Available from:

For peer review only - http://bmjopen.bmj.com/site/about/guidelines.xhtml

https://pubmed.ncbi.nlm.nih.gov/27059888

4. Sands R, Shanmugavadivel D, Stephenson T, Wood D. Medical problems presenting to paediatric emergency departments: 10 years on. Emerg Med J [Internet]. 2012 May 1;29(5):379 LP – 382. Available from: http://emj.bmj.com/content/29/5/379.abstract

5. Van Den Bruel A, Bartholomeeusen S, Aertgeerts B, Truyers C, Buntinx F. Serious infections in children: An incidence study in family practice. BMC Fam Pract. 2006;7:1–9.

6. Riordan FAI, Thomson APJ. Recognition, Treatment and Complications of Meningococcal Disease. Pediatr Drugs [Internet]. 1999;1(4):263–82. Available from: https://doi.org/10.2165/00128072-199901040-00003

7. Van Hecke O, Raymond M, Lee JJ, Turner P, Goyder CR, Verbakel JY, et al. In-vitro diagnostic point-of-care tests in paediatric ambulatory care: A systematic review and meta-analysis. PLoS One [Internet]. 2020;15(7). Available from: http://dx.doi.org/10.1371/journal.pone.0235605

8. Bruel A Van den, Haj-Hassan T, Thompson M, Buntinx F, Mant D. Diagnostic value of clinical features at presentation to identify serious infection in children in developed countries: a systematic review. Lancet [Internet]. 2010 Mar 6 [cited 2020 Aug 14];375(9717):834–45. Available from: https://www.thelancet.com/journals/lancet/article/PIIS0140-6736(09)62000-6/fulltext#.XzZBwQcE2mc.mendeley

9. Gill PJ, Goldacre MJ, Mant D, Heneghan C, Thomson A, Seagroatt V, et al. Increase in emergency admissions to hospital for children aged under 15 in England, 1999-2010: National database analysis. Arch Dis Child. 2013;98(5):328–34.

10. Saxena S, Bottle A, Gilbert R, Sharland M. Increasing short-stay unplanned hospital admissions among children in England; Time trends analysis '97-'06. PLoS One. 2009;4(10):1–6.

11. Cecil E, Bottle A, Sharland M, Saxena S. Impact of UK primary care policy reforms on shortstay unplanned hospital admissions for children with primary care–sensitive conditions. Ann Fam Med. 2015;13(3):214–20.

12. Wyatt S, Mohammed MA, Fisher E, McConkey R, Spilsbury P. Impact of the SARS-CoV-2 pandemic and associated lockdown measures on attendances at emergency departments in English hospitals: A retrospective database study. Lancet Reg Heal - Eur [Internet]. 2021;2:100034. Available from: https://doi.org/10.1016/j.lanepe.2021.100034

13. Hartshorn S, O'Sullivan R, Maconochie IK, Bevan C, Cleugh F, Lyttle MD. Establishing the research priorities of paediatric emergency medicine clinicians in the UK and Ireland. Emerg Med J. 2015;32(11):864–8.

14. Miller SZ, Rincón H, Kuppermann N, (PECARN) the PECARN. Revisiting the Emergency Medicine Services for Children Research Agenda: Priorities for Multicenter Research in Pediatric Emergency Care. Acad Emerg Med [Internet]. 2008 Apr 1;15(4):377–83. Available from: https://doi.org/10.1111/j.1553-2712.2008.00072.x

15. Bialy L, Plint AC, Freedman SB, Johnson DW, Curran JA, Stang AS, et al. Pediatric Emergency Research Canada (PERC): Patient/Family-informed Research Priorities for Pediatric Emergency Medicine. Acad Emerg Med [Internet]. 2018 Dec 1;25(12):1365–74. Available from: https://doi.org/10.1111/acem.13493

16. Jones CHD, Howick J, Roberts NW, Price CP, Heneghan C, Plüddemann A, et al.

For peer review only - http://bmjopen.bmj.com/site/about/guidelines.xhtml

Primary care clinicians' attitudes towards point-of-care blood testing: A systematic review of qualitative studies. BMC Fam Pract. 2013;14.

17. Candidate 111003, Dewez M, Yeung S. MSc Project Report The Perceptions of English Paediatricians on the Use of Point-of-Care-Tests (POCTs) in Assessing Febrile Children . 2018;(September):1–50.

18. Candidate 110875, Dewez M, Yeung S. MSc Project Report Perceptions of English Emergency Department Healthcare Providers on the Use of Rapid Diagnostic Tests (RDTs) in Febrile Children: A Qualitative Study. 2018;(September):1–88.

19. Patton MQ. How to use qualitative methods in evaluation . Newbury Park, Calif: Sage Publications; 1987. (Program evaluation kit (2nd ed.) ; 4).

20. Strauss A, Corbin J. Basics of Qualitative Research: Techniques and Procedures for Developing Grounded Theory. 2nd ed. Thousand Oaks, CA: Sage; 1990.

21. Durand MA, Chantler T, editors. Principles of social research . Second edi. Maidenhead, England: Open University Press; 2014. (Understanding Public Health series).

22. NHS. Integrating Care: [Internet]. Integrating Care: Next steps to building strong and effective integrated care systems across England. 2021. Available from: https://www.england.nhs.uk/publication/integrating-care-next-steps-to-building-strong-and-effective-integrated-care-systems-across-england/

23. Anderson M, Pitchforth E, Asaria M, Brayne C, Casadei B, Charlesworth A, et al. LSE–Lancet Commission on the future of the NHS: re-laying the foundations for an equitable and efficient health and care service after COVID-19. Lancet [Internet]. 2021;397(10288):1915–78. Available from: https://www.sciencedirect.com/science/article/pii/S0140673621002324

24. Panicker L. Nurses' Perceptions of Parent Empowerment in Chronic Illness. Contemp Nurse. 2013 May 26;45:210–9.

25. Van Den Bruel A, Jones C, Thompson M, Mant D. C-reactive protein point-of-care testing in acutely ill children: A mixed methods study in primary care. Arch Dis Child. 2016;101(4):382–6.

For peer review only

For peer review only - http://bmjopen.bmj.com/site/about/guidelines.xhtml

Table 1 Complete participant characteristics

Participant	Job role	Time in that role /years (mo= months)	Gender	Level 1= primary 2= secondary 3= tertiary	Setting Rural 0 Urban 1	Recruitment 1= PERUKI 2=Website 3=Direct 4=Snowball 5=conference
Clinicians						
01	Consultant paediatric and neonatal surgeon<br>BAPS	5<br>2	M	3<br>n/a	1	3
02	Consultant paediatrician	2	M	2	0	1
03	GP	1 mo	F	1	1	4
04	Specialist asthma nurse	26	F	1	1	4
05	Macmillan GP<br>CCG clinical lead for children and young people	12<br>5 mo	F	1<br>n/a	1	3
06	Consultant in paediatric and adult emergency medicine	14	F	2	1	1
07	Specialist paediatric trainee	5	F	2	1	4
08	Foundation Year 1 doctor	2 mo	M	2	1	4
09	GP<br>CCG clinical lead for cancer, children & maternity	20<br>8	M	1<br>n/a	1	2
10	GP	4	F	1	1	5
11	Consultant children's orthopaedic surgeon	4	M	3	1	3
12	Primary care advanced nurse practitioner	35	F	1	0	4
13	Consultant community paediatrician	21	M	2	Mixture	3
14	Consultant community psychiatrist of children and adolescents	3.5	F	2	Mixture	3
15	Senior staff nurse children's emergency department	4	F	3	1	3
16	Urgent care GP	18	M	1	1	2
17	Primary care advanced nurse practitioner	23	F	1	1	3
Stakeholders						

For peer review only - http://bmjopen.bmj.com/site/about/guidelines.xhtml

Participant	Job role	Time in that role /years (mo= months)	Gender	Level 1= primary 2= secondary 3= tertiary	Setting Rural 0 Urban 1	Recruitment 1= PERUKI 2=Website 3=Direct 4=Snowball 5=conference
01	Meningitis Research Foundation	2	F	n/a	n/a	3
02	Little Miracles	10	F	n/a	n/a	3
03	Asthma UK<br>GP	3<br>15	M	n/a<br>1	Mixture	3
04	HappyR health	1	M	n/a	n/a	3
05	CCG clinical lead for children, young people & maternity<br>GP	20<br>7	F	n/a<br>1	1	3

BAPS, British Association of Paediatric Surgeons, GP General Practitioner, CCG Clinical Commissioning Group, PERUKI Paediatric Emergency Research in the UK and Ireland. N/A Not Applicable. Participants highlighted in grey are both clinicians and stakeholders.

Table 2 Main themes and subthemes

Theme 1: Potential benefits of POC tests and technologies
1a: POC tests facilitate early decision-making
1b: Home-based POC tests are convenient
1c: POC tests are empowering for children and their families
Theme 2: Areas for improvement for POC tests and technologies
2a: POC tests should be more widely available
2b: End-users should find POC tests quick and easy to use
2c: POC tests should be agreeable and engaging for children
2d: POC tests should make a difference to clinical management

For peer review only - http://bmjopen.bmj.com/site/about/guidelines.xhtml

Table 3 Additional participant quotes listed by theme and sub-theme

Theme	Sub-theme	Test/technology	Quote	Participant
**1: Potential benefits of POC tests and technologies**	1a POC tests facilitate early decision-making	Spirometry, FeNO	*"Tests, such as, spirometry and FeNO are good objective measures which we can use at the bedside to help decide whether… somebody has or doesn't have asthma… a lot of patients get under diagnosed… that means they're getting chronic symptoms and inflammation and ongoing damage within the airways… which can cause… disability from stopping them doing normal things in their life; it can put them at risk of life-threatening asthma attacks and it can cause chronic inflammation of the lungs causing long-term damage."*	Stakeholder#3
	1b: Home-based POC tests are convenient	Remote observations	*"from a patient perspective and a practice perspective… seeing as much as we can remotely is… much better. Nobody in their right mind wants to bring a sick child out and sit in a doctor's surgery waiting for a doctor or practitioner to be running late [when] the kid's not well"*	Clinician#17
**2: Areas for improvement for POC tests and technologies**	2b: End-users should find POC tests quick and easy to use	Urinalysis	*"we had an example of a [teenage] girl… with fairly non-specific symptoms… Had not been able to produce the urine, said they would do it later, that didn't happen… the diagnosis was made about perhaps a week later [of] diabetes"*	Clinician#12
		Smart inhaler	*"there is one device that clips to one specific inhaler… it measures the sound of the inhalation so you can gauge whether or not… that dose has been taken properly… currently it's only being used in research, but the potential is there"*	Clinician#4
		Monitoring of exhaled gases	*" before long there will be the technology that when you talk into your mobile phone it will be able to monitor your asthma… a combined exhaled carbon monoxide and nitric oxide monitor"*	Clinician#4

POC point-of-care, FeNO Fractional exhaled Nitric Oxide

For peer review only

Table 4 Unmet needs: Ideas for application of new tests or technologies that have not already been mentioned in Table 3

Test/technology/pathway	Quote	Participant
**Acute serious illness**		
Predicting severity of bronchiolitis	*"it's really difficult to tell which, which babies are going to have a mild broncholitic course and just settle down quite quickly and those that are going to progress and need additional respiratory support, so… whether there's a breath-activated test… that tells you… [that] would be incredible"*	Clinician#2
Remote observations using smartphone cameras and apps	*"we… are wary of sepsis for example…. in children who are poorly with acute illnesses we… spend quite a lot of time gaining information about those particular sepsis markers so I will be checking their oxygen levels, I'll be measuring their respiratory rate. I'll be checking their pulse. I'll be checking their blood pressure if that's appropriate. We'll be checking their temperature, their capillary refill time… if a patient could do that [at home] so there is an App which can [quickly and non-invasively] assess these [sepsis] markers… that would be hugely helpful… in making a decision safely…and may mean that less patients need to be assessed face to face or in hospital… it would save us a lot of time and would provide a lot of assistance"*	Clinician#15
Poisons and seizures	*"you can send the blood test off and get paracetamol salicylate levels; that's fairly standard… It would be helpful to get those results earlier [with] other drugs… for your older teenager who comes in unconscious and you're wondering what they might have taken…. children with epilepsy… are they taking the right dose of sodium valproate?... if you could find that out quickly then would, that would change our management… when they're coming in having a seizure"*	Clinician#6
Appendicitis	*"if you had a child who was suspected to have appendicitis clinically, but you wanted to be more certain, then you would have access to… a bedside ultrasound… and prove definitively whether they did or did not… 1) it could provide better selection of children who needed to have treatment for their appendicitis; and 2)… it could give reassurance to those who didn't have appendicitis so they could be sent home"*	Clinician#1
Ovarian torsion	*"ultrasound is used for ovarian torsion… [that] could be done at the bedside"*	Clinician#1
Fracture	*"avoiding X-rays, doing near patient ultrasound to diagnose your fracture or whatever it is. … some of this stuff can really help with minors, reducing radiation exposure of children and, and speeding up the process"*	Clinician#6
**Distinguishing bacterial and viral infection**		
Diagnosing bacterial meningitis	*"you could distinguish viral meningitis and bacterial meningitis to high sensitivity and specificity with this 2 RNA transcript signature"*	Stakeholder#1
	"I have read about the rapid DNA test for Neisseria meningitis… and that will be very useful in the context of a child presenting with non-blanching rash and fever… I tend to over treat these kind of children or to admit for observations waiting for… blood tests to come back"	Clinician#7
**Diagnosing and monitoring chronic disease**		
Assessing pain or stress in children unable to communicate	*"kids with ASD … you could monitor where [and] when their heart rate goes up and when there's more signs of stress, even if they don't realise that they're getting stressed at these times… some objective monitoring could be helpful for those kids because they're not very aware of their own emotions...you can [then] plan an intervention accordingly".*	Clinician#14
Diagnosing genetic diseases	*"we're talking of whole genetic sequencing coming along very, very quickly now…getting the results by the bedside"*	Clinician#13

RNA, ribonucleic acid; ASD, autistic spectrum disorder

For peer review only

For peer review only - http://bmjopen.bmj.com/site/about/guidelines.xhtml

Standards for Reporting Qualitative Research: A Synthesis of Recommendations

Bridget C. O'Brien, PhD, Ilene B. Harris, PhD, Thomas J. Beckman, MD, Darcy A. Reed, MD, MPH, and David A. Cook, MD, MHPE

Abstract

Purpose
Standards for reporting exist for many types of quantitative research, but currently none exist for the broad spectrum of qualitative research. The purpose of the present study was to formulate and define standards for reporting qualitative research while preserving the requisite flexibility to accommodate various paradigms, approaches, and methods.

Method
The authors identified guidelines, reporting standards, and critical appraisal criteria for qualitative research by searching PubMed, Web of Science, and Google through July 2013; reviewing the reference lists of retrieved sources; and contacting experts. Specifically, two authors reviewed a sample of sources to generate an initial set of items that were potentially important in reporting qualitative research. Through an iterative process of reviewing sources, modifying the set of items, and coding all sources for items, the authors prepared a near-final list of items and descriptions and sent this list to five external reviewers for feedback. The final items and descriptions included in the reporting standards reflect this feedback.

Results
The Standards for Reporting Qualitative Research (SRQR) consists of 21 items. The authors define and explain key elements of each item and provide examples from recently published articles to illustrate ways in which the standards can be met.

Conclusions
The SRQR aims to improve the transparency of all aspects of qualitative research by providing clear standards for reporting qualitative research. These standards will assist authors during manuscript preparation, editors and reviewers in evaluating a manuscript for potential publication, and readers when critically appraising, applying, and synthesizing study findings.

Qualitative research contributes to the literature in many disciplines by describing, interpreting, and generating theories about social interactions and individual experiences as they occur in natural, rather than experimental, situations.[1–3] Some recent examples include studies of professional dilemmas,[4] medical students' early experiences of workplace learning,[5] patients' experiences of disease and interventions,[6–8] and patients' perspectives about incident disclosures.[9] The purpose of qualitative research is to understand the perspectives/experiences of individuals or groups and the contexts in which these perspectives or experiences are situated.[1,2,10]

Please see the end of this article for information about the authors.

Correspondence should be addressed to Dr. O'Brien, Office of Research and Development in Medical Education, UCSF School of Medicine, Box 3202, 1855 Folsom St., Suite 200, San Francisco, CA 94143-3202; e-mail: bridget.obrien@ucsf.edu.

Acad Med. 2014;89:1245–1251.
First published online June 20, 2014
doi: 10.1097/ACM.0000000000000388

Supplemental digital content for this article is available at http://links.lww.com/ACADMED/A218.

Qualitative research is increasingly common and valued in the medical and medical education literature.[1,10–13] However, the quality of such research can be difficult to evaluate because of incomplete reporting of key elements.[14,15] *Quality* is multifaceted and includes consideration of the importance of the research question, the rigor of the research methods, the appropriateness and salience of the inferences, and the clarity and completeness of reporting.[16,17] Although there is much debate about standards for methodological rigor in qualitative research,[13,14,18–20] there is widespread agreement about the need for clear and complete reporting.[14,21,22] Optimal reporting would enable editors, reviewers, other researchers, and practitioners to critically appraise qualitative studies and apply and synthesize the results. One important step in improving the quality of reporting is to formulate and define clear reporting standards.

Authors have proposed guidelines for the quality of qualitative research, including those in the fields of medical education,[23–25] clinical and health services research,[26–28] and general education research.[29,30] Yet in nearly all cases, the authors do not describe how the guidelines were created, and often fail to distinguish reporting quality from the other facets of quality (e.g., the research question or methods). Several authors suggest standards for reporting qualitative research,[15,20,29–33] but their articles focus on a subset of qualitative data collection methods (e.g., interviews), fail to explain how the authors developed the reporting criteria, narrowly construe qualitative research (e.g., thematic analysis) in ways that may exclude other approaches, and/or lack specific examples to help others see how the standards might be achieved. Thus, there remains a compelling need for defensible and broadly applicable standards for reporting qualitative research.

We designed and carried out the present study to formulate and define standards for reporting qualitative research through a rigorous synthesis of published articles and expert recommendations.

Method

We formulated standards for reporting qualitative research by using a rigorous and systematic approach in which we reviewed previously proposed

For peer review only

Downloaded from http://journals.lww.com/academicmedicine by BhDMf5ePHKav1zEoum1tQfN4a+kJLhEZgbsIHo4XMi0hCywCX1AWnYQp/IlQrHD3i3D0OdRyi7TvSFl4Cf3VC4/OAVpDDa8K2+Ya6H515kE= on 11/08/2021

For peer review only - http://bmjopen.bmj.com/site/about/guidelines.xhtml

recommendations by experts in qualitative methods. Our research team consisted of two PhD researchers and one physician with formal training and experience in qualitative methods, and two physicians with experience, but no formal training, in qualitative methods.

We first identified previously proposed recommendations by searching PubMed, Web of Science, and Google using combinations of terms such as "qualitative methods," "qualitative research," "qualitative guidelines," "qualitative standards," and "critical appraisal" and by reviewing the reference lists of retrieved sources, reviewing the Equator Network,[22] and contacting experts. We conducted our first search in January 2007 and our last search in July 2013. Most recommendations were published in peer-reviewed journals, but some were available only on the Internet, and one was an interim draft from a national organization. We report the full set of the 40 sources reviewed in Supplemental Digital Appendix 1, found at http://links.lww.com/ACADMED/A218.

Two of us (B.O., I.H.) reviewed an initial sample of sources to generate a comprehensive list of items that were potentially important in reporting qualitative research (Draft A). All of us then worked in pairs to review all sources and code the presence or absence of each item in a given source. From Draft A, we then distilled a shorter list (Draft B) by identifying core concepts and combining related items, taking into account the number of times each item appeared in these sources. We then compared the items in Draft B with material in the original sources to check for missing concepts, modify accordingly, and add explanatory definitions to create a prefinal list of items (Draft C).

We circulated Draft C to five experienced qualitative researchers (see the acknowledgments) for review. We asked them to note any omitted or redundant items and to suggest improvements to the wording to enhance clarity and relevance across a broad spectrum of qualitative inquiry. In response to their reviews, we consolidated some items and made minor revisions to the wording of labels and definitions to create the final set of reporting standards—the Standards for Reporting Qualitative Research (SRQR)—summarized in Table 1.

To explicate how the final set of standards reflect the material in the original sources, two of us (B.O., D.A.C.) selected by consensus the 25 most complete sources of recommendations and identified which standards reflected the concepts found in each original source (see Table 2).

Results

The SRQR is a list of 21 items that we consider essential for complete, transparent reporting of qualitative research (see Table 1). As explained above, we developed these items through a rigorous synthesis of prior recommendations and concepts from published sources (see Table 2; see also Supplemental Digital Appendix 1, found at http://links.lww.com/ACADMED/A218) and expert review. These 21 items provide a framework and recommendations for reporting qualitative studies. Given the wide range of qualitative approaches and methodologies, we attempted to select items with broad relevance.

The SRQR includes the article's title and abstract (items 1 and 2); problem formulation and research question (items 3 and 4); research design and methods of data collection and analysis (items 5 through 15); results, interpretation, discussion, and integration (items 16 through 19); and other information (items 20 and 21). Supplemental Digital Appendix 2, found at http://links.lww.com/ACADMED/A218, contains a detailed explanation of each item, along with examples from recently published qualitative studies. Below, we briefly describe the standards, with a particular focus on those unique to qualitative research.

Titles, abstracts, and introductory material. Reporting standards for titles, abstracts, and introductory material (problem formulation, research question) in qualitative research are very similar to those for quantitative research, except that the results reported in the abstract are narrative rather than numerical, and authors rarely present a specific hypothesis.[29,30]

Research design and methods. Reporting on research design and methods of data collection and analysis highlights several distinctive features of qualitative research. Many of the criteria we reviewed focus not only on identifying and describing all aspects of the methods (e.g., approach, researcher characteristics and role, sampling strategy, context, data collection and analysis) but also on justifying each choice.[13,14] This ensures that authors make their assumptions and decisions transparent to readers. This standard is less commonly expected in quantitative research, perhaps because most quantitative researchers share positivist assumptions and generally agree about standards for rigor of various study designs and sampling techniques.[14] Just as quantitative reporting standards encourage authors to describe how they implemented methods such as randomization and measurement validity, several qualitative reporting criteria recommend that authors describe how they implemented a presumably familiar technique in their study rather than simply mentioning the technique.[10,14,32] For example, authors often state that data collection occurred until saturation, with no mention of how they defined and recognized saturation. Similarly, authors often mention an "iterative process," with minimal description of the nature of the iterations. The SRQR emphasizes the importance of explaining and elaborating on these important processes. Nearly all of the original sources recommended describing the characteristics and role of the researcher (i.e., reflexivity). Members of the research team often form relationships with participants, and analytic processes are highly interpretive in most qualitative research. Therefore, reviewers and readers must understand how these relationships and the researchers' perspectives and assumptions influenced data collection and interpretation.[15,23,26,34]

Results. Reporting of qualitative research results should identify the main analytic findings. Often, these findings involve interpretation and contextualization, which represent a departure from the tradition in quantitative studies of objectively reporting results. The presentation of results often varies with the specific qualitative approach and methodology; thus, rigid rules for reporting qualitative findings are inappropriate. However, authors

For peer review only

Table 1

Standards for Reporting Qualitative Research (SRQR)[a]

No.	Topic		Item
	**Title and abstract**		
S1	Title	✓ p1	Concise description of the nature and topic of the study Identifying the study as qualitative or indicating the approach (e.g., ethnography, grounded theory) or data collection methods (e.g., interview, focus group) is recommended
S2	Abstract	✓ p1	Summary of key elements of the study using the abstract format of the intended publication; typically includes background, purpose, methods, results, and conclusions
	**Introduction**		
S3	Problem formulation	✓ 3-4	Description and significance of the problem/phenomenon studied; review of relevant theory and empirical work; problem statement
S4	Purpose or research question	✓ p4	Purpose of the study and specific objectives or questions
	**Methods**		
S5	Qualitative approach and research paradigm	✓ p4	Qualitative approach (e.g., ethnography, grounded theory, case study, phenomenology, narrative research) and guiding theory if appropriate; identifying the research paradigm (e.g., postpositivist, constructivist/ interpretivist) is also recommended; rationale[b]
S6	Researcher characteristics and reflexivity	✓ p5	Researchers' characteristics that may influence the research, including personal attributes, qualifications/experience, relationship with participants, assumptions, and/or presuppositions; potential or actual interaction between researchers' characteristics and the research questions, approach, methods, results, and/or transferability
S7	Context	✓ p4	Setting/site and salient contextual factors; rationale[b]
S8	Sampling strategy	p4 ✓	How and why research participants, documents, or events were selected; criteria for deciding when no further sampling was necessary (e.g., sampling saturation); rationale[b]
S9	Ethical issues pertaining to human subjects	p5 ✓	Documentation of approval by an appropriate ethics review board and participant consent, or explanation for lack thereof; other confidentiality and data security issues
S10	Data collection methods	p5-6 ✓	Types of data collected; details of data collection procedures including (as appropriate) start and stop dates of data collection and analysis, iterative process, triangulation of sources/methods, and modification of procedures in response to evolving study findings; rationale[b]
S11	Data collection instruments and technologies	p4-5 ✓	Description of instruments (e.g., interview guides, questionnaires) and devices (e.g., audio recorders) used for data collection; if/how the instrument(s) changed over the course of the study
S12	Units of study	p6, Table 1 ✓	Number and relevant characteristics of participants, documents, or events included in the study; level of participation (could be reported in results)
S13	Data processing	p5 ✓	Methods for processing data prior to and during analysis, including transcription, data entry, data management and security, verification of data integrity, data coding, and anonymization/deidentification of excerpts
S14	Data analysis	p5 ✓	Process by which inferences, themes, etc., were identified and developed, including the researchers involved in data analysis; usually references a specific paradigm or approach; rationale[b]
S15	Techniques to enhance trustworthiness	p5 ✓	Techniques to enhance trustworthiness and credibility of data analysis (e.g., member checking, audit trail, triangulation); rationale[b]
	**Results/findings**		
S16	Synthesis and interpretation	6-10 ✓	Main findings (e.g., interpretations, inferences, and themes); might include development of a theory or model, or integration with prior research or theory
S17	Links to empirical data	6-9 ✓	Evidence (e.g., quotes, field notes, text excerpts, photographs) to substantiate analytic findings
	**Discussion**		
S18	Integration with prior work, implications, transferability, and contribution(s) to the field	p3, 10 ✓	Short summary of main findings; explanation of how findings and conclusions connect to, support, elaborate on, or challenge conclusions of earlier scholarship; discussion of scope of application/ generalizability; identification of unique contribution(s) to scholarship in a discipline or field
S19	Limitations	p3 ✓	Trustworthiness and limitations of findings

(*Table continues*)

For peer review only

Table 1
(Continued)

No.	Topic			Item
	**Other**			
S20	Conflicts of interest	p11	✓	Potential sources of influence or perceived influence on study conduct and conclusions; how these were managed
S21	Funding	p11	✓	Sources of funding and other support; role of funders in data collection, interpretation, and reporting

[a]The authors created the SRQR by searching the literature to identify guidelines, reporting standards, and critical appraisal criteria for qualitative research; reviewing the reference lists of retrieved sources; and contacting experts to gain feedback. The SRQR aims to improve the transparency of all aspects of qualitative research by providing clear standards for reporting qualitative research.
[b]The rationale should briefly discuss the justification for choosing that theory, approach, method, or technique rather than other options available, the assumptions and limitations implicit in those choices, and how those choices influence study conclusions and transferability. As appropriate, the rationale for several items might be discussed together.

For peer review only

should provide evidence (e.g., examples, quotes, or text excerpts) to substantiate the main analytic findings.[20,29]

Discussion. The discussion of qualitative results will generally include connections to existing literature and/or theoretical or conceptual frameworks, the scope and boundaries of the results (transferability), and study limitations.[10–12,28] In some qualitative traditions, the results and discussion may not have distinct boundaries; we recommend that authors include the substance of each item regardless of the section in which it appears.

Discussion

The purpose of the SRQR is to improve the quality of reporting of qualitative research studies. We hope that these 21 recommended reporting standards will assist authors during manuscript preparation, editors and reviewers in evaluating a manuscript for potential publication, and readers when critically appraising, applying, and synthesizing study findings. As with other reporting guidelines,[35–37] we anticipate that the SRQR will evolve as it is applied and evaluated in practice. We welcome suggestions for refinement.

Qualitative studies explore "how?" and "why?" questions related to social or human problems or phenomena.[10,38] Purposes of qualitative studies include understanding meaning from participants' perspectives (How do they interpret or make sense of an event, situation, or action?); understanding the nature and influence of the context surrounding events or actions; generating theories about new or poorly understood events, situations, or actions; and understanding the processes that led to a desired (or undesired) outcome.[38] Many different approaches (e.g., ethnography, phenomenology, discourse analysis, case study, grounded theory) and methodologies (e.g., interviews, focus groups, observation, analysis of documents) may be used in qualitative research, each with its own assumptions and traditions.[1,2] A strength of many qualitative approaches and methodologies is the opportunity for flexibility and adaptability throughout the data collection and analysis process. We endeavored to maintain that flexibility by intentionally defining items to avoid favoring one approach or method over others. As such, we trust that the SRQR will support all approaches and methods of qualitative research by making reports more explicit and transparent, while still allowing investigators the flexibility to use the study design and reporting format most appropriate to their study. It may be helpful, in the future, to develop approach-specific extensions of the SRQR, as has been done for guidelines in quantitative research (e.g., the CONSORT extensions).[37]

Limitations, strengths, and boundaries

We deliberately avoided recommendations that define methodological rigor, and therefore it would be inappropriate to use the SRQR to judge the quality of research methods and findings. Many of the original sources from which we derived the SRQR were intended as criteria for methodological rigor or critical appraisal rather than reporting; for these, we inferred the information that would be needed to evaluate the criterion. Occasionally, we found conflicting recommendations in the literature (e.g., recommending specific techniques such as multiple coders or member checking to demonstrate trustworthiness); we resolved these conflicting recommendations through selection of the most frequent recommendations and by consensus among ourselves.

Some qualitative researchers have described the limitations of checklists as a means to improve methodological rigor.[13] We nonetheless believe that a checklist for reporting standards will help to enhance the transparency of qualitative research studies and thereby advance the field.[29,39]

Strengths of this work include the grounding in previously published criteria, the diversity of experience and perspectives among us, and critical review by experts in three countries.

Implications and application

Similar to other reporting guidelines,[35–37] the SRQR may be viewed as a starting point for defining reporting standards in qualitative research. Although our personal experience lies in health professions education, the SRQR is based on sources originating in diverse health care and non-health-care fields. We intentionally crafted the SRQR to include various paradigms, approaches, and methodologies used in qualitative research. The elaborations offered in

For peer review only - http://bmjopen.bmj.com/site/about/guidelines.xhtml

Table 2

Alignment of the 21 Standards for Reporting Qualitative Research (SRQR) With Recommendations From 25 Original Sources[a]

		Reference no.[b]																							
**No.**	**Topic**	**11,12**	**15[c]**	**19**	**20[c]**	**23**	**24,25[d]**	**26**	**27**	**29[c,d]**	**30[c,d]**	**31[c]**	**32[c]**	**33**	**34**	**41**	**42**	**43**	**44[c]**	**45**	**46**	**47**	**48**	**49**	**50**
S1	Title						*	*		*														*	
S2	Abstract						*			*	*			*											
S3	Problem formulation				*	*	*	*	*	*	*	*		*	*	*	*	*			*			*	*
S4	Purpose or research question	*	*	*	*	*	*	*	*	*	*	*	*	*	*	*	*	*		*	*	*	*	*	*
S5	Qualitative approach and research paradigm	*	*	*	*	*	*	*		*	*		*	*		*	*	*			*	*	*	*	*
S6	Researcher characteristics, reflexivity	*	*	*	*	*	*	*	*	*		*	*	*		*	*	*	*	*	*	*	*	*	*
S7	Context		*	*	*	*	*	*	*	*	*	*		*		*	*		*	*	*		*		*
S8	Sampling strategy	*	*	*	*	*	*	*	*	*	*	*	*	*	*	*	*	*	*	*	*		*	*	
S9	Ethical issues pertaining to human subjects	*			*		*			*	*		*	*		*	*	*		*	*	*			*
S10	Data collection methods	*	*	*	*	*	*	*	*	*	*	*	*	*	*	*	*	*	*	*	*	*	*	*	*
S11	Data collection instruments/ technologies	*	*				*			*	*	*	*	*		*		*			*				
S12	Units of study	*	*		*		*	*		*	*	*	*	*		*					*			*	
S13	Data processing	*				*	*	*		*	*	*	*				*				*				*
S14	Data analysis	*	*	*	*	*	*	*	*	*	*	*	*	*	*	*	*	*	*	*	*	*	*	*	*
S15	Techniques to enhance trustworthiness	*	*	*	*	*	*	*	*	*	*	*	*	*	*	*	*	*	*	*	*		*	*	*
S16	Synthesis and interpretation	*	*		*	*	*	*	*	*	*	*	*	*		*	*	*			*	*	*	*	*
S17	Links to empirical data	*	*		*	*	*	*	*	*	*	*		*		*	*	*	*			*	*		*
S18	Integration with prior work, implications, transferability, and contribution(s)	*		*	*	*	*	*	*	*	*	*	*	*		*	*	*	*	*	*		*	*	*
S19	Limitations	*			*	*	*	*		*				*		*	*	*			*				*
S20	Conflicts of interest						*			*															
S21	Funding									*						*								*	

[a]The authors created the SRQR by searching the literature to identify guidelines, reporting standards, and critical appraisal criteria for qualitative research; reviewing the reference lists of retrieved sources; and contacting experts to gain feedback. The SRQR aims to improve the transparency of all aspects of qualitative research by providing clear standards for reporting qualitative research. In the table, the asterisks indicate which sources mentioned which topics.
[b]The numbers in column headings are the numbers of the citations in the reference list at the end of this report. Those citations are of original sources describing criteria for reporting and/or critical appraisal of qualitative research, which the authors used in creating the SRQR.
[c]Focuses on reporting standards (all other sources focus on quality standards or guidelines for critical review/evaluation).
[d]Addresses quantitative and qualitative research.

Supplemental Digital Appendix 2 (see http://links.lww.com/ACADMED/A218) should provide sufficient description and examples to enable both novice and experienced researchers to use these standards. Thus, the SRQR should apply broadly across disciplines, methodologies, topics, study participants, and users.

For peer review only

For peer review only - http://bmjopen.bmj.com/site/about/guidelines.xhtml

The SRQR items reflect information essential for inclusion in a qualitative research report, but should not be viewed as prescribing a rigid format or standardized content. Individual study needs, author preferences, and journal requirements may necessitate a different sequence or organization than that shown in Table 1. Journal word restrictions may prevent a full exposition of each item, and the relative importance of a given item will vary by study. Thus, although all 21 standards would ideally be reflected in any given report, authors should prioritize attention to those items that are most relevant to the given study, findings, context, and readership.

Application of the SRQR need not be limited to the writing phase of a given study. These standards can assist researchers in planning qualitative studies and in the careful documentation of processes and decisions made throughout the study. By considering these recommendations early on, researchers may be more likely to identify the paradigm and approach most appropriate to their research, consider and use strategies for ensuring trustworthiness, and keep track of procedures and decisions.

Journal editors can facilitate the review process by providing the SRQR to reviewers and applying its standards, thus establishing more explicit expectations for qualitative studies. Although the recommendations do not address or advocate specific approaches, methods, or quality standards, they do help reviewers identify information that is missing from manuscripts.

As authors and editors apply the SRQR, readers will have more complete information about a given study, thus facilitating judgments about the trustworthiness, relevance, and transferability of findings to their own context and/or to related literature. Complete reporting will also facilitate meaningful synthesis of qualitative results across studies.[40] We anticipate that such transparency will, over time, help to identify previously unappreciated gaps in the rigor and relevance of research findings. Investigators, editors, and educators can then work to remedy these deficiencies and, thereby, enhance the overall quality of qualitative research.

Acknowledgments: The authors thank Margaret Bearman, PhD, Calvin Chou, MD, PhD, Karen Hauer, MD, Ayelet Kuper, MD, DPhil, Arianne Teherani, PhD, and participants in the UCSF weekly educational scholarship works-in-progress group (ESCape) for critically reviewing the Standards for Reporting Qualitative Research.

Funding/Support: This study was funded in part by a research review grant from the Society for Directors of Research in Medical Education.

Other disclosures: None reported.

Ethical approval: Reported as not applicable.

Disclaimer: The funding agency had no role in the study design, analysis, interpretation, writing of the manuscript, or decision to submit the manuscript for publication.

Dr. O'Brien is assistant professor, Department of Medicine and Office of Research and Development in Medical Education, University of California, San Francisco, School of Medicine, San Francisco, California.

Dr. Harris is professor and head, Department of Medical Education, University of Illinois at Chicago College of Medicine, Chicago, Illinois.

Dr. Beckman is professor of medicine and medical education, Department of Medicine, Mayo Clinic College of Medicine, Rochester, Minnesota.

Dr. Reed is associate professor of medicine and medical education, Department of Medicine, Mayo Clinic College of Medicine, Rochester, Minnesota.

Dr. Cook is associate director, Mayo Clinic Online Learning, research chair, Mayo Multidisciplinary Simulation Center, and professor of medicine and medical education, Mayo Clinic College of Medicine, Rochester, Minnesota.

References

1 Lingard L, Kennedy TJ. Qualitative research in medical education. In: Swanwick T, ed. Understanding Medical Education: Evidence, Theory and Practice. Oxford, UK: Wiley-Blackwell; 2010:323–335.
2 Harris IB. Qualitative methods. In: Norman GR, van der Vleuten CPM, Newble DI, eds. International Handbook of Research in Medical Education. Dordrecht, Netherlands: Kluwer Academic Publishers; 2002:45–95.
3 Denzin N, Lincoln Y. Introduction: The discipline and practice of qualitative research. In: The Sage Handbook of Qualitative Research. 3rd ed. Thousand Oaks, Calif: Sage Publications, Inc.; 2005:1–32.
4 Ginsburg S, Bernabeo E, Ross KM, Holmboe ES. "It depends": Results of a qualitative study investigating how practicing internists approach professional dilemmas. Acad Med. 2012;87:1685–1693.
5 Yardley S, Brosnan C, Richardson J, Hays R. Authentic early experience in medical education: A socio-cultural analysis identifying important variables in learning interactions within workplaces. Adv Health Sci Educ Theory Pract. 2013;18:873–891.
6 Embuldeniya G, Veinot P, Bell E, et al. The experience and impact of chronic disease peer support interventions: A qualitative synthesis. Patient Educ Couns. 2013;92:3–12.
7 Pinnock H, Kendall M, Murray SA, et al. Living and dying with severe chronic obstructive pulmonary disease: Multi-perspective longitudinal qualitative study. BMJ. 2011;342:d142.
8 Brady MC, Clark AM, Dickson S, Paton G, Barbour RS. Dysarthria following stroke: The patient's perspective on management and rehabilitation. Clin Rehabil. 2011;25:935–952.
9 Iedema R, Allen S, Britton K, et al. Patients' and family members' views on how clinicians enact and how they should enact incident disclosure: The "100 patient stories" qualitative study. BMJ. 2011;343:d4423.
10 Kuper A, Reeves S, Levinson W. An introduction to reading and appraising qualitative research. BMJ. 2008;337:404–407.
11 Giacomini M, Cook, DJ. Users' guides to the medical literature: XXIII. Qualitative research in health care A. Are the results of the study valid? JAMA. 2000;284:357–362.
12 Giacomini M, Cook, DJ. Users' guides to the medical literature: XXIII. Qualitative research in health care B. What are the results and how do they help me care for my patients? JAMA. 2000;284:478–482.
13 Barbour RS. Checklists for improving rigour in qualitative research: A case of the tail wagging the dog? BMJ. 2001;322:1115–1117.
14 Dunt D, McKenzie R. Improving the quality of qualitative studies: Do reporting guidelines have a place? Fam Pract. 2012;29:367–369.
15 Tong A, Sainsbury P, Craig J. Consolidated criteria for reporting qualitative research (COREQ): A 32-item checklist for interviews and focus groups. Int J Qual Health Care. 2007;19:349–357.
16 Cook DA, Bowen JL, Gerrity MS, et al. Proposed standards for medical education submissions to the Journal of General Internal Medicine. J Gen Intern Med. 2008;23:908–913.
17 Tracy SJ. Qualitative quality: Eight "big-tent" criteria for excellent qualitative research. Qual Inq. 2010;16:837–851.
18 Lincoln YS. Emerging criteria for quality in qualitative and interpretive research. Qual Inq. 1995;1:275–289.
19 Mays N, Pope C. Qualitative research in health care. Assessing quality in qualitative research. BMJ. 2000;320:50–52.
20 Burns N. Standards for qualitative research. Nurs Sci Q. 1989;2:44–52.
21 Ryan GW. What Are Standards of Rigor for Qualitative Research? 2005. http://www.wjh.harvard.edu/nsfqual/Ryan%20Paper.pdf. Accessed April 20, 2014.
22 The EQUATOR Network: Enhancing the quality and transparency of health research. http://www.equator-network.org. Accessed April 6, 2014.
23 Côté L, Turgeon J. Appraising qualitative research articles in medicine and medical education. Med Teach. 2005;27:71–75.
24 Bordage G, Caelleigh AS. A tool for reviewers: "Review criteria for research manuscripts." Acad Med. 2001;76:904–951.
25 Task Force of Academic Medicine and the GEA-RIME Committee. Appendix 1: Checklist of review criteria. Acad Med. 2001;76:958–959.
26 Malterud K. Qualitative research: Standards, challenges, and guidelines. Lancet. 2001;358:483–488.

For peer review only - http://bmjopen.bmj.com/site/about/guidelines.xhtml

27 Inui TS, Frankel RM. Evaluating the quality of qualitative research: A proposal pro tem. J Gen Intern Med. 1991;6:485–486.
28 Devers KJ. How will we know" good" qualitative research when we see it? Beginning the dialogue in health services research. Health Serv Res. 1999;34:1153.
29 Duran RP, Eisenhart MA, Erickson FD, et al. Standards for reporting on empirical social science research in AERA publications. Educ Res. 2006;35:33–40.
30 Newman M, Elbourne D. Improving the usability of educational research: Guidelines for the reporting of primary empirical research studies in education (The REPOSE Guidelines). Eval Res Educ. 2004;18:201–212.
31 Knafl KA, Howard MJ. Interpreting and reporting qualitative research. Res Nurs Health. 1984;7:17–24.
32 Kitto SC, Chesters J, Grbich C. Quality in qualitative research. Med J Aust. 2008;188:243–246.
33 Rowan M, Huston P. Qualitative research articles: Information for authors and peer reviewers. CMAJ. 1997;157:1442–1446.
34 Cohen D, Crabtree B. Guidelines for designing, analyzing, and reporting qualitative research. Qualitative Research Guidelines Project, Robert Wood Johnson Foundation. 2006. http://qualres.org/HomeGuid-3868.html. Accessed April 6, 2014.
35 Elm E von, Altman DG, Egger M, Pocock SJ, Gøtzsche PC, Vandenbroucke JP. Strengthening the reporting of observational studies in epidemiology (STROBE) statement: Guidelines for reporting observational studies. BMJ. 2007;335:806–808.
36 Davidoff F, Batalden P, Stevens D, Ogrinc G, Mooney S; SQUIRE Development Group. Publication guidelines for quality improvement in health care: Evolution of the SQUIRE project. Qual Saf Health Care. 2008;17(suppl 1):i3–i9.
37 Schulz KF, Altman DG, Moher D; CONSORT Group. CONSORT 2010 statement: Updated guidelines for reporting parallel group randomised trials. BMJ. 2010;340:c332.
38 Maxwell JA. Designing a qualitative study. In: Bickman L, Bog D, eds. The SAGE Handbook of Applied Social Research Methods. 2nd ed. Sage Publications, Inc.; 2009:214–253.
39 Meyrick J. What is good qualitative research? A first step towards a comprehensive approach to judging rigour/quality. J Health Psychol. 2006;11:799–808.
40 Bearman M, Dawson P. Qualitative synthesis and systematic review in health professions education. Med Educ. 2013;47:252–260.

References Cited Only in Table 2

41 Attree P, Milton B. Critically appraising qualitative research for systematic reviews. Evid Policy. 2006;2:109–126.
42 Blaxter M. Criteria for the evaluation of qualitative research. Med Sociol News. 1996;22:34–37.
43 Critical Appraisal Skills Programme (CASP). Qualitative Research Checklist. 2013. http://www.casp-uk.net/wp-content/uploads/2011/11/casp-qualitative-research-checklist-31.05.13.pdf#!casp-tools-checklists/c18f8. Accessed April 6, 2014.
44 Frambach JM, van der Vleuten CP, Durning SJ. AM last page. Quality criteria in qualitative and quantitative research. Acad Med. 2013;88:552.
45 Kuper A, Lingard L, Levinson W. Critically appraising qualitative research. BMJ. 2008;337:687–689.
46 Law M, Stewart D, Letts L, Pollock N, Bosch J, Westmorland M. Guidelines for the critical review of qualitative studies. McMaster University Occupational Therapy Evidence-Based Practice Research Group. 1998. http://www.usc.edu/hsc/ebnet/res/Guidelines.pdf. Accessed April 20, 2014.
47 Pearson A, Field J, Jordan Z. Appendix 2: Critical appraisal tools. In: Evidence-Based Clinical Practice in Nursing and Health Care: Assimilating Research, Experience and Expertise. Oxford, UK: Blackwell Publishing Ltd.; 2009:177–182. http://onlinelibrary.wiley.com/doi/10.1002/9781444316544.app2/summary. Accessed April 13, 2014.
48 Popay J, Rogers A, Williams G. Rationale and standards for the systematic review of qualitative literature in health services research. Qual Health Res. 1998;8:341–351.
49 Sandelowski M, Barroso J. Writing the proposal for a qualitative research methodology project. Qual Health Res. 2003;13:781–820.
50 Stige B, Malterud K, Midtgarden T. Toward an agenda for evaluation of qualitative research. Qual Health Res. 2009;19:1504–1516.

For peer review only

For peer review only - http://bmjopen.bmj.com/site/about/guidelines.xhtml

BMJ Open

Point-of-care Diagnostic Technology in Paediatric Ambulatory Care: a Qualitative Interview study of English Clinicians and Stakeholders

Journal:	*BMJ Open*
Manuscript ID	bmjopen-2021-059103.R1
Article Type:	Original research
Date Submitted by the Author:	17-Mar-2022
Complete List of Authors:	Raymond, Meriel; University of Oxford, Nuffield Department of Primary Care Health Sciences<br>Bird, Chris; Birmingham Children's Hospital, Emergency Department; University of Oxford Nuffield Department of Primary Care Health Sciences<br>van Hecke, Oliver; Oxford University, Nuffield Department of Primary Care Health Sciences<br>Glogowska, Margaret; Oxford University, Nuffield Department of Primary Care Health Sciences<br>Hayward, Gail; Oxford University, Nuffield Department of Primary Care Health Sciences
**Primary Subject Heading**:	Diagnostics
Secondary Subject Heading:	Paediatrics
Keywords:	Molecular diagnostics < INFECTIOUS DISEASES, Paediatric A&E and ambulatory care < PAEDIATRICS, QUALITATIVE RESEARCH, PRIMARY CARE

SCHOLARONE™
Manuscripts

I, the Submitting Author has the right to grant and does grant on behalf of all authors of the Work (as defined in the below author licence), an exclusive licence and/or a non-exclusive licence for contributions from authors who are: i) UK Crown employees; ii) where BMJ has agreed a CC-BY licence shall apply, and/or iii) in accordance with the terms applicable for US Federal Government officers or employees acting as part of their official duties; on a worldwide, perpetual, irrevocable, royalty-free basis to BMJ Publishing Group Ltd ("BMJ") its licensees and where the relevant Journal is co-owned by BMJ to the co-owners of the Journal, to publish the Work in this journal and any other BMJ products and to exploit all rights, as set out in our licence.

The Submitting Author accepts and understands that any supply made under these terms is made by BMJ to the Submitting Author unless you are acting as an employee on behalf of your employer or a postgraduate student of an affiliated institution which is paying any applicable article publishing charge ("APC") for Open Access articles. Where the Submitting Author wishes to make the Work available on an Open Access basis (and intends to pay the relevant APC), the terms of reuse of such Open Access shall be governed by a Creative Commons licence – details of these licences and which Creative Commons licence will apply to this Work are set out in our licence referred to above.

Other than as permitted in any relevant BMJ Author's Self Archiving Policies, I confirm this Work has not been accepted for publication elsewhere, is not being considered for publication elsewhere and does not duplicate material already published. I confirm all authors consent to publication of this Work and authorise the granting of this licence.

For peer review only

Point-of-care Diagnostic Technology in Paediatric Ambulatory Care: a Qualitative Interview study of English Clinicians and Stakeholders

Dr. Meriel Raymond*,
Dr. Chris Bird,
Dr. Oliver Van Hecke,
Dr. Margaret Glogowska,
Professor Gail Hayward

Nuffield Department of Primary Care Health Sciences,
University of Oxford, Oxford, UK

*Corresponding author. Tel: +44(0)1865-289300

E-mail: meriel.raymond@phc.ox.ac.uk orcid.org/0000-0002-0442-9280

Keywords
Bedside
Diagnostics
Paediatrics
Ambulatory care facilities
Qualitative

For peer review only

Abstract

Point-of-care (POC) tests have the potential to improve paediatric healthcare. However, both the development and evaluation of POC technology have almost solely been focussed on adults. We aimed to explore frontline clinicians' and stakeholders' current experience of POC diagnostic technology in children in England; and to identify areas of unmet need.

Design, setting and participants

Qualitative semi-structured telephone interviews were carried out with purposively sampled participants from clinical paediatric ambulatory care and charity, industry and policymaking stakeholders. The interviews were audio-recorded, transcribed and analysed thematically.

Results

We interviewed 19 clinicians and eight stakeholders. The main perceived benefits of POC tests and technologies were that they aided early decision-making and could be convenient and empowering when used independently by patients and families. Clinicians and stakeholders wanted more POC tests to be available for use in clinical practice. Most recognised that play and reward are important components of successful POC tests for children. Clinicians wanted tests to give them answers which would result in a change in their clinical management. Detecting acute serious illness, notably distinguishing viral and bacterial infection, was perceived to be an area where tests could add value. POC tests were thought to be particularly useful for children presenting atypically, where diagnosis was more challenging, such as those less able to communicate, and for rare serious diseases. Many participants felt they could be useful in managing chronic disease.

Conclusions

This exploratory study found that clinicians and stakeholders supported the use of diagnostic POC technology in paediatric ambulatory care settings in England. Some existing tests are not fit for purpose and could be refined. Industry should be encouraged to develop new child-friendly tests tackling areas of unmet need, guided by the preferred characteristics of those working on the ground.

For peer review only - http://bmjopen.bmj.com/site/about/guidelines.xhtml

Article Summary

Strengths and limitations of this study

- Semi-structured interviews enabled an in-depth exploration of the experiences of the heterogeneous participants with different backgrounds.
- Purposive sampling with snowballing facilitated the interviewing of a broad range of clinicians and stakeholders on this topic. Inclusion of stakeholders enabled emergence of views from policymaking and industry perspectives.
- However, the broad remit of the study meant that we were unable to cover every single test and paediatric clinical presentation, making “data saturation” difficult to achieve.
- Although children’s and parents’ perspectives were mentioned by our participants, and some offered their own experiences as parents; their views were not specifically sought in this study.
- All participants were based in England. As such our findings are applicable to English stakeholders and clinicians and may not be transferable to other settings.

Introduction

Paediatric ambulatory care places huge demand on healthcare services. One in four consultations in ambulatory care in the UK are for children [1,2]. Children present with a different disease spectrum to adults, having a high incidence of acute infections [3]. Most of these consultations are for upper respiratory tract infections which are generally self-limiting. The incidence of serious infection in children presenting to primary care has been estimated to be less than 1% [3]. The challenge in primary care is that these serious infections often present with non-specific symptoms, especially in the early stages. Furthermore, children have the potential to deteriorate more quickly than adults [4]. It is difficult to detect those children who will progress to serious illness requiring secondary care input [7] in a timely way [5]. Inappropriate prescribing, unnecessary referrals to hospital and needless additional testing often result from this diagnostic uncertainty [6]. There was a 10–20% trend increase in potentially avoidable, short stay hospital admissions of children in England from 1997–2012 [7–9]. The onset the SARS-CoV-2 pandemic in March-April 2020 saw 69% less children attending emergency departments in the UK [10]; this was followed by a 1-4% increase in attendance per week. Paediatric emergency research groups have identified the need to develop better diagnostics for “low numbers, high stakes diagnoses” in children [11–13].

POC (point-of-care) tests can be defined as any test performed near a patient or clinic with results available during a clinical visit [14,15]. Point-of-care technology includes measurements taken at the bedside, such as smartphone applications and wearables. POC tests have the potential to reduce diagnostic uncertainty in acute illness and streamline management of chronic disease, improving clinical outcomes and reducing health-related costs [5]. A systematic review and meta-analysis of the clinical impact of POC tests in paediatric ambulatory care found few studies [5]. The use of malarial POC tests was found to reduce over-treatment by a third compared to usual care. HIV-POC tests improved early initiation of antiretroviral therapy compared to usual care. POC C-reactive protein may reduce immediate antibiotic prescribing for respiratory tract infections in low-and-middle-

For peer review only - http://bmjopen.bmj.com/site/about/guidelines.xhtml

income countries, but evidence was lacking in high-income countries. The evaluation of POC tests for children often lags behind that for adults, for example with SARS-CoV-2 testing [16].

Attitudes of primary care clinicians towards POC blood tests in Europe and Australia have been synthesised in one systematic review of qualitative studies [14]. Participants thought that POC testing improved diagnostic certainty, treatment, self-management of chronic disease, clinician-patient relationships, and perceived patient experience. The views of English paediatricians and Emergency Department healthcare providers on the use of POC tests to assess febrile children have also been explored [17]. This study agreed with previous publications on POC tests' advantages – improved patient flow, quicker decision making, minimal invasiveness of testing and improved antibiotic stewardship – but also had concerns about a decrease in clinical acumen, the reliability of POC tests and the issue that some POC tests with a continuous variable made clinical decision making more, not less, difficult. This paper suggested seeking the views of paediatricians in district general hospitals, GPs (general practitioners), and other paediatric subspecialities.

Other recent studies have highlighted obstacles to greater use of POC tests in children. Pandey et al, in a survey of UK children's Emergency Departments and paediatric assessment units, found lack of funding, a lack of evidence, and governance issues surrounding quality assurance of tests meant several new biomarkers which already exist had not been adopted in the majority of units [18]. Rasti et al, in a qualitative survey of nurses and doctors in a Swedish children's emergency department, found that while POC tests' benefits included better satisfaction from families who wanted a test for their child and greater reassurance in some instances in clinical decision making, those surveyed feared the use of POC tests in hospital and at home might drive more unnecessary testing and that reliance on POC tests could diminish clinical skills [19].

Little is known about attitudes of primary care clinicians towards POC tests more broadly than blood tests in children. There is little information on stakeholders' views; or views towards POC technologies, including apps and wearables.

The diagnostic needs in paediatric ambulatory care are unlikely to be met by diagnostics which have been developed with an adult population primarily in mind. Children are not "mini adults" and have specific needs that should be addressed in order for diagnostics to be helpful in a clinical setting. These might include the requirement for rapid diagnosis, smaller sample volumes and less invasive procedures. Point-of-care (POC) tests have the potential to address these needs. In order to stimulate the development and evaluation of POC diagnostic technology which is of greatest benefit in paediatric healthcare it is important to understand the current experience of those using these technologies and identify areas of unmet need. We aimed to seek the views and experiences of a broad range of clinicians and stakeholders with an interest in paediatric ambulatory care in the UK about current usage and unmet needs for POC diagnostic technology.

Methods

Qualitative research is highly appropriate for capturing and exploring people's experiences and perceptions; and has considerable power to explain actions, decisions and processes [20]. Therefore, qualitative interviews were used to explore perceptions of clinicians and stakeholders towards POC tests and technologies in paediatric ambulatory care.

For peer review only - http://bmjopen.bmj.com/site/about/guidelines.xhtml

Sampling and recruitment

A maximum variation, purposive sample of participants was sought based on gender, level of clinical experience, and range of NHS settings [21]. We advertised for participants using the PERUKI (Paediatric Emergency Research in the UK and Ireland) mailing list in August 2019 and April 2020, and on the website for the Nuffield Department of Primary Care Health Sciences (NDPCHS), University of Oxford, from 19th June 2019 at www.phc.ox.ac.uk/iTAP.

We directly approached specialist clinicians, children's commissioners, CCGs (Clinical Commissioning Groups; groups of general practices which come together in each area to commission services for their patients and population), children's charities pertaining to serious illness, and TITCH (Technology Innovation Transforming Child Health) using telephone or email details that were in the public domain.

Recruitment was extended to contacts of participants in a "snowballing" effect. Early interviews shaped the identification of further interviewees, using a principle of grounded theory; namely, theoretical sampling which permits the deliberate inclusion of participants whose viewpoints have been shown to be of interest [22]. The decision to stop interviewing, when sufficient information had emerged and there was satisfactory explanation for the emerging themes, was discussed and agreed among the research team.

Interviews

Qualitative semi-structured individual interviews were conducted by the primary researcher MR. These enabled in-depth exploration of the experiences of the heterogeneous participants [23], through interviewer and interviewee interaction, and exploration of details which were significant to either party as the interview progressed. A focus group discussion of a wide range of professionals would be less likely to capture these individual experiences. Focus-group discussion was also avoided due to logistical difficulty in arranging group clinician sessions; need for HRA (Health Research Authority) approval for interviews occurring on NHS (National Health Service) premises; and divergence of stakeholder interests.

Participants were offered a telephone or face-to-face interview of around 30 minutes. Due to participant preference and the COVID-19 pandemic, all interviews were conducted by telephone. Informed verbal consent was obtained prior to interview. Draft topic guides for the interviews with clinicians and stakeholders were developed to address the study objectives (see Supplementary Materials). These were based on available literature, and drew on issues from topic guides for other studies we have conducted around clinicians' views of POC testing [24,25]. The topic guide was initially reviewed by the research team; modified iteratively by the primary researcher based on feedback; and amended after 12 interviews following discussion with the research team. Participants were informed "by point-of-care tests and technologies, we mean any diagnostic technology to include tests on bodily fluids, imaging, wearables, digital technology, and smart phone apps". Interviews were recorded using a digital audio-recorder and transcribed verbatim by a single professional transcriber. Field notes were made by the primary researcher during and after the interviews. Data were stored and processed in line with GDPR (General Data Protection Regulation). In recognition of the time contributed to the study, interviewed participants were offered a £20 gift voucher.

For peer review only

Analysis

Transcripts were anonymised and checked against the audio recordings for accuracy. Anonymised transcripts were uploaded into a specialist software programme to assist organisation of data (NVivo version 12). A "ground up" approach from the data was adopted to analyse the complete data set [26] using thematic analysis [23]. The primary researcher read and familiarised herself with the transcripts. Systematic and detailed codes were compared and grouped to create categories. These were organised into an initial "data driven" coding framework based on six coded interviews. These interviews were read by MG and GH and the coding framework checked. This coding framework was iteratively applied to subsequent transcripts. "Constant comparison" was used to cross-check ideas and categories that were emerging across interviews, taking an inductive approach [20]. Broad themes were developed using "single sheet" brainstorming [20]. Agreement on coding, themes, and subthemes, was sought between members of the research team. An audit trail from the raw data of the interview transcripts through coding to development of themes was established to ensure dependability. Participants were provided with the results section and given two weeks to provide feedback.

Researcher characteristics and reflexivity

The primary researcher was a General Practitioner undertaking a master's degree in public health. She attended a course on Qualitative Interviewing prior to the study. The participants were aware of her clinical background prior to interview and her reasons for undertaking the research. MG is a specialist qualitative researcher.

Ethics

Ethical approval was obtained from the Medical Sciences Interdivisional Research Ethics Committee, University of Oxford, on the 30th April 2019 (reference R63109/RE001); and LSHTM (London School of Hygiene & Tropical Medicine) MSc Research Ethics Committee on the 14th May 2019 (reference 17436).

Public and patient involvement

No patients were involved. The final manuscript was sent to participants.

Results

22 interviews were conducted between June 2019 and July 2020. The interviews lasted an average of 35 minutes.

Participant characteristics

For complete participant characteristics please see Table 1. Of the 22 participants, 14 were clinicians; three stakeholders; and five were both clinicians and stakeholders.
Of the 19 clinicians, nine were from primary care (seven GPs, two nurses), and ten from secondary or tertiary care (eight doctors, two nurses).
The eight stakeholders represented three CCGs (Clinical Commissioning Groups), three charities, and one Tech Company.

For peer review only - http://bmjopen.bmj.com/site/about/guidelines.xhtml

Themes and sub-themes

The main themes and sub-themes are described below in Table 2**Table 2** Main themes and subthemes .

Theme 1: Potential benefits of POC tests and technologies

1a: POC tests facilitate early decision-making

Participants reported that the predominant advantage of POC tests and technologies is that they give rapid results compared to tests requiring laboratory processing or transfer of the child to another department. They thought that POC tests increased the speed of clinicians' decisions and allowed the assessing clinicians to incorporate the result as part of their holistic assessment. Delayed laboratory results would be more likely to be interpreted by a clinician who had not seen the child.

"you don't really know if this lump is an abscess or not, which can guide your treatment and management; having to rely on a radiologist really delays the treatment of the child and makes you… admit the child for the scan to happen the next day... …if you had the chance to do that by the bedside… that….would really make a difference [Emergency Department Consultant Clinician#6]

A Macmillan GP (GP with palliative care as a specialist interest) thought that availability of POC full blood count in primary care settings would facilitate faster pick-up of difficult-to-diagnose serious conditions such as childhood cancer, as a delay in hospital referral often delayed the diagnosis.

"they'd been back and forwards to the GP with tiredness or a bit of a viral infection… and it was only when they got into A&E [Accident and Emergency]… that the blood tests [were] done and the leukaemia was found… probably a barrier for us in primary [care] at the moment is that we would have to refer the patient to… the hospital… but if we could just do it in primary care that probably would… transform that sort of diagnosis". [Macmillan GP, Clinician#5]

Many clinicians and stakeholders thought that POC technologies could help to give earlier diagnosis of chronic disease, enabling prompt appropriate treatment and decreasing morbidity. Examples were given of spirometry and Fractional Exhaled Nitric Oxide (FeNO) [see Table 3]; POC eosinophils; and mental health questionnaires.

Clinicians and stakeholders representing children with additional needs, disabilities and life limiting conditions, added that early pick-up of clinical deterioration was particularly important, as they often had an *"up and down trajectory and a high risk of sudden episodes of acute illness"* [GP Clinician#5]. They thought it might be worth monitoring such children at home to pick up early physiological changes as a "*safety net*" [GP Clinician#5].

1b: Home-based POC tests are convenient

Participants suggested that POC tests performed at home by patients and their families or caregivers could decrease the need for face-to-face assessment in health care settings. An example was given of the use of POC clotting testing in children with replacement heart valves "*improv[ing] the quality of those families' lives*" making a "*really big difference*" [Community paediatrician Clinician#13]. Participants felt that home testing would be

For peer review only - http://bmjopen.bmj.com/site/about/guidelines.xhtml

convenient for patients and clinicians and could speed up recognition and escalation of acute illness. Furthermore, it was thought that this would improve infection prevention and control, particularly during the COVID-19 pandemic. An unmet need was identified for the detection of vital signs including temperature and oxygen levels by parents at home, for example with smart-phone cameras (see Table 3).

1c: POC tests are empowering for children and their families

Participants explained that the additional objective information given by POC tests and technologies to children and their families would empower them to communicate their illness more effectively to health care professionals, facilitating the consultation. This was particularly important for the families or carers of children who struggled to communicate because of disability, and in whom detection of illness is more difficult.

"families find communication about a problem with healthcare services quite challenging and if they were equipped with a range of clinical parameters to help their discussion… they might find they access the right kind of healthcare quicker" [GP Clinician#5]

Furthermore, participants said that the results from these tests helped children with chronic disease and their families to look after their own health better.

"I have heard of young people using and parents taking control of diabetes management using Apps quite pro-actively…….[they attend] clinic and consultants [feel] a bit redundant because suddenly they've been replaced by this App which is giving their family a lot more control… [they] are actually making those decision themselves about management…we can… empower people to actually self-manage these conditions very effectively" [GP Clinician#5]

Theme 2: Areas for improvement for POC tests and technologies

2a: POC tests should be more widely available

Most of the participants had not come across many POC technologies in their clinical practice, or felt that were not widely available. They also thought that cost, for example of FeNO and peripheral oxygen saturation monitors, could limit accessibility and lead to "*inequitable distribution*" [Asthma nurse Clinician#4].

2b: End-users should find POC tests quick and easy to use

Many participants felt that POC tests and technologies need to be quick to use, so that a child could be distracted, for instance during a distressing test; or not lose concentration, for instance during measurement of peak flow. The "*time-poor*" clinicians [GP Clinician#9] also wanted quick tests; firstly to improve patient flow, and secondly to enable continuity, in that the same clinician seeing the patient at initial contact could also be responsible for interpreting the result. Some participants expressed a preference for tests that would give results in seconds. Innovations they suggested included contactless scanning to measure oxygen saturations and height [Emergency Department nurse Clinician #15]; measurement of basic observations with smartphone cameras [GP Clinician #16] or use of smartphone apps to diagnose rashes [Advanced nurse practitioner Clinician#12].

Participants reported that POC tests need to be easy to perform to avoid causing pain and stress for children and their families. This was particularly true for finger pricks, throat swabs and blood pressure measurements. There was however a consensus that finger prick tests

For peer review only - http://bmjopen.bmj.com/site/about/guidelines.xhtml

using a single drop of blood are acceptable. Many participants stated that urine samples (see Table 3), peak flows and spirometry could be challenging for younger children to perform. Participants said that POC tests and technologies requiring no extra effort by the child would be ideal (see Table 3, smart inhaler and monitoring of exhaled gases).

Many participants felt that tests and technologies needed to be "*fool proof*" to perform [Emergency Department Consultant Clinician#6]. Participants reported that where tests were not easy to use, it put them off using them. They frequently gave the example of measuring peripheral oxygen saturations which posed a logistical challenge in primary care as it was often difficult to obtain a reliable result. One participant stated *"there's a gap of a non-single-use [oxygen saturation] probe that is effective and quick to use"* [Advanced nurse practitioner Clinician#17].

"With younger kids… under five years of age… and particularly babies under one… we've got one [Peripheral oxygen saturations monitor] machine per practice. So first of all, I have to go out and get it, find the box. It might be… in the right place or maybe another clinician's got it. You've got to send a message out, "Who's got the [oxygen saturations] machine?"… it seems to take… four or five minutes sometimes to get a reading. You fidget around, try on the thumb… end up trying earlobes and things… it's just really hard when, on young babies you try across the foot and the kid starts wriggling and kicking… and then if you're unlucky you'll get a bad trace and… it's not actually their sats because the pulse rate's completely wrong… but if it starts to then blip and say things like 80 per cent, you just start thinking, 'Oh God, why the hell did I do this" [GP Clinician#10]

2c: POC tests should be agreeable and engaging for children

Many participants felt that POC tests should ideally be enjoyable. The asthma nurse [Clinician#4] described making peak flows into a game. Reward was particularly important in children with disability.

"anything that could be done as a wearable, so that… they're still able to play. A lot of the kids that we have when they go into A&E, they might be really quite poorly but actually… it's usual for them… They just want to be able to play and… get on with their life…. ….and so it's then quite inconvenient and they get upset… and quite angry and quite stroppy… because… it's interfering with their day… anything that we can do to… make it less medicalised and more play-based, more fun [is] always a good thing". [Little Miracles Stakeholder#2]

Visual results such as FeNO were described as engaging the patient and increasing adherence with medication. When children entered information into one stakeholder's app, their progress was indicated by the growth of a plant [Stakeholder#4].

"FeNO is massively useful in patients that are… not adherent with their medication in that it gives them that lightbulb moment to actually visualise what's going on inside the chest… [if] you can then illustrate that by measuring an inflammatory marker, they tend to be a bit more adherent". [Asthma nurse Clinician#4]

2d: POC tests should make a difference to clinical management

Participants wanted POC tests and technologies to give them results that would make a difference to their decision-making and get them *"further ahead"* [Emergency Department

For peer review only - http://bmjopen.bmj.com/site/about/guidelines.xhtml

Consultant Clinician #6]. They felt that "*something objective*" [GP Clinician#10] might "*stop interpersonal and intrapersonal variance"* [Paediatrician Clinician#2]. Many of them expressed a wish for tests with "*good sensitivity and specificity to be reliable*" [Foundation Year 1 Doctor (junior doctor in their first year of practice) Clinician#8]. Participants wanted confirmatory tests to enable detection of acute serious illness *"to rule out the worst-case scenario"* [Paediatric trainee Clinician#7]. For instance, many clinicians asserted that low peripheral oxygen saturations would help pick up acute serious illness, and guide referral to hospital, mode of transport to hospital, and need for admission. A GP [Clinician #9] had invested £500 in a machine because of this perceived impact. One participant [Paediatrician Clinician#2] felt that these basic observations were sometimes under-utilised in the clinical setting, and that this could be a focus for improvement over the development of new tests or technologies.

"I sometimes don't recognise that people are as bad as they are because I'm a bit too optimistic. But sometimes I'll see a child… and say, 'Actually, you don't look…too bad' and then I'll put the oximetry on and go… 'Oh, actually, you're worse than I realised. Let's just think about this a bit more seriously'" [GP Clinician #9]

The acute serious illnesses that participants raised were predominantly sepsis and meningitis, with an emphasis on the need to distinguish between bacterial and viral infection, and confirmation of a specific pathogen being particularly helpful. This could increase clinician confidence in diagnosis and management, including antibiotic prescribing. They gave examples of POC streptococcal PCR and POC respiratory PCR panels in primary care.

"URTI {Upper Respiratory Tract Infection}-type symptoms… the research nurse did [nasopharyngeal swabs] and they could run the analyser and within an hour you would know whether this had a bacterial element to it and then obviously you could prescribe [antibiotics] if that was appropriate… the parents [had] such a willingness to take part in that research trial… the fact that you could say to them, 'Yeah we can test you straight away now,' and we can get an answer to you… parents were very happy with that" [Advanced Nurse Practitioner Clinician#12]

The importance of exact pathogen detection in the context of public health was also raised, with implications for contact-tracing and vaccination when meningococci and SARS-CoV-2 were detected. Participants acknowledged that results might offer false reassurance, for example in a viral respiratory tract infection, and that clinicians would still need to safety net against development of a secondary bacterial infection. Desire for POC tests to assist in diagnosis of non-infective acute serious illness including ischaemia, diabetes, cancer, seizures, poisoning and trauma were also mentioned in the interviews; as were tests to diagnose chronic disease such as asthma and genetic conditions. Suggestions for areas of innovation are listed with quotes in Table 4.

Discussion

Summary of main findings

There are areas of unmet need for POC tests in paediatric ambulatory care. Participants wanted more POC tests and technologies to be available. They thought they should be user-friendly and, where possible, fun. They felt that they could empower patients and their families when used at home; particularly in children with chronic disease. Clinicians wanted

For peer review only - http://bmjopen.bmj.com/site/about/guidelines.xhtml

POC tests to give results that made a difference to clinical management; especially in the detection of acute serious illness in children for whom diagnosis is more challenging.

Strengths and weaknesses of this study

Strengths of this study include the use of semi-structured interviews, enabling an in-depth exploration of the experiences of the heterogeneous participants with different backgrounds [23]. Purposive sampling with snowballing facilitated the interviewing of a broad range of clinicians and stakeholders on this topic. The participants had diverse job roles, work settings and levels of experience. This enabled a wide variety of perspectives to be captured including those from policymaking and industry. Important needs of particular groups of children were highlighted because specialist experts were purposively sampled.

However, the broad remit of the study meant that we were unable to cover every single test and paediatric clinical presentation, making “data saturation” [20] difficult to achieve. Understanding of specific POC tests, as well as specific clinical presentations and contexts, could be examined in a more in-depth way in a focused study. Furthermore, although children’s and parents’ perspectives were mentioned by our participants, and some offered their own experiences as parents, their views were not specifically sought in this study. Finally, all participants were based in England. As such our findings are applicable to English stakeholders and clinicians and may not be transferable to other settings.

Findings in relation to other studies

Our finding of unmet needs corroborated one systematic meta-analysis which demonstrated that very few studies, limited to a handful of diseases, have shown benefit of POC tests in paediatric populations [5]. Concerns over lack of funding were similarly found in a survey of UK children’s emergency departments and paediatric assessment units [18]In keeping with the concerns expressed in that survey about quality assurance, our participants stated that they wanted tests with high specificity and sensitivity. In contrast to that survey, our participants did not express concern that there was lack of evidence surrounding the use of POC tests.

Our study also shared some findings with a qualitative systematic review assessing clinicians’ attitudes towards POC blood tests in primary care settings in high income countries [14]. For example, many of our participants thought that POC tests could facilitate early clinical decision-making, as did the clinicians in the systematic review. In our study, participants placed new importance on the use of POC tests and technologies for earlier detection of acute serious illness in children who present atypically, and for whom diagnosis is normally delayed as a result.

Our study highlighted that the convenient use of POC tests at home by patients and their families could bypass the need for clinician assessment and empower patients and families. This is in keeping with the NHS’s promotion of Integrated Care Systems [27], and development of better diagnostics to improve diagnostic bottlenecks and help tackle health inequalities [28]. Child health nurses have highlighted in an interview study that parents felt empowered by being able to take care of their child in a safe and structured way at home [29].Our participants didn’t express the concern found in a Swedish study of hospital clinicians that POC testing at home may drive unnecessary testing [19].

For peer review only - http://bmjopen.bmj.com/site/about/guidelines.xhtml

The preference of our participants for POC tests to be easy to use and avoid causing pain was also evident in a more focussed interview study of English hospital clinicians [17]. Their belief that finger prick testing is acceptable has similarly been demonstrated in GP settings [30]. Our study highlighted new information that play, visualisation and reward are important components of successful POC tests and technologies in children.

Many of our participants wanted tests that would make a difference to clinical management- particularly to flag risk of serious clinical deterioration; and distinguish between viral and bacterial disease. This was also found by a qualitative study of English hospital healthcare workers [17]. Both that study and our study have raised the importance of particular pathogen testing for infection control- theirs RSV; ours SARS-CoV-2 and meningococcus. Many of our participants expressed a preference for panels of pathogens, as did the first study.

Implications for clinicians, policymakers, and industry

We found that UK clinicians and stakeholders were of the opinion that existing bedside tests were not fit-for-purpose in ambulatory care paediatrics. One priority should be refining and enhancing existing tests, for example the measurement of oxygen saturations in young children.

Participants wanted POC tests to be routinely available in clinical practice with the potential for tests to be used by children and their carers at home. For diagnostic developers, our study offers evidence in favour of the design of POC tests and technologies that incorporate play and reward to make them more acceptable to children and their carers.

Unanswered questions and future research

Further qualitative and health services research to evaluate preferred characteristics of POC tests and technologies from parents and children themselves is advised to guide future “patient-up” development by industry. This study highlighted that this would be particularly important in children who present atypically such as children with disability, and children diagnosed with cancer. This would enable more equitable representation of children with greater healthcare needs.

A variety of unmet needs for diagnostics in paediatric ambulatory care were identified by our study, such as reliable early detection of acute serious illness, and the ‘holy grail’ of differentiation between viral and bacterial illness. This provides support for investment in research and development in these areas.

Word Count

4869 words

Acknowledgements

We acknowledge our participants who took the time to participate in our study.

For peer review only - http://bmjopen.bmj.com/site/about/guidelines.xhtml

Author contributions

MR- Interviews, Project administration, Data curation, Formal analysis, Writing – original draft, Writing – review & editing
CB- Writing – review & editing
OVH- Writing – review & editing
MG- Conceptualization, Methodology, Supervision, Writing – review & editing
GH- Conceptualization, Funding acquisition, Methodology, Supervision, Writing – review & editing

Data sharing statement

Participants gave their permission for the sharing of anonymised data. This will be subject to appropriate research requests, screened by a panel of the authorship team.

Funding

This research was funded by the National Institute for Health Research (NIHR) Community Healthcare MedTech and in Vitro Diagnostics Co-operative (CH MIC), award code MIC-2016-018. MR's salary was funded by an In-practice fellowship grant from the NIHR.

Transparency declarations

MR affirms that the manuscript is an honest, accurate and transparent account of the study being reported, that no important aspects of the study have been omitted, and that any discrepancies from the study as planned have been explained.

Disclaimer

The views expressed are those of the authors and not necessarily those of the NHS, the NIHR or the Department of Health and Social Care.

Competing interest

All authors have completed the ICMJE uniform disclosure form at http://www.icmje.org/disclosure-of-interest/ and declare: all authors had financial support from NIHR CH MIC for the submitted work; no financial relationships with any organisations that might have an interest in the submitted work in the previous three years; no other relationships or activities that could appear to have influenced the submitted work.

References

1 Hobbs FDR, Bankhead C, Mukhtar T, *et al.* Clinical workload in UK primary care: a retrospective analysis of 100 million consultations in England, 2007-14. *Lancet* 2016;**387**:2323–30. doi:10.1016/S0140-6736(16)00620-6

2 Sands R, Shanmugavadivel D, Stephenson T, *et al.* Medical problems presenting to paediatric emergency departments: 10 years on. *Emergency Medicine Journal* 2012;**29**:379 LP – 382. doi:10.1136/emj.2010.106229

3 Van Den Bruel A, Bartholomeeusen S, Aertgeerts B, *et al.* Serious infections in children: An incidence study in family practice. *BMC Family Practice* 2006;**7**:1–9. doi:10.1186/1471-2296-7-23

For peer review only - http://bmjopen.bmj.com/site/about/guidelines.xhtml

4 Riordan FAI, Thomson APJ. Recognition, Treatment and Complications of Meningococcal Disease. *Pediatric Drugs* 1999;**1**:263–82. doi:10.2165/00128072-199901040-00003

5 Van Hecke O, Raymond M, Lee JJ, *et al.* In-vitro diagnostic point-of-care tests in paediatric ambulatory care: A systematic review and meta-analysis. *PLoS ONE* 2020;**15**. doi:10.1371/journal.pone.0235605

6 Bruel A Van den, Haj-Hassan T, Thompson M, *et al.* Diagnostic value of clinical features at presentation to identify serious infection in children in developed countries: a systematic review. *The Lancet* 2010;**375**:834–45. doi:10.1016/S0140-6736(09)62000-6

7 Saxena S, Bottle A, Gilbert R, *et al.* Increasing short-stay unplanned hospital admissions among children in England; Time trends analysis '97-'06. *PLoS ONE* 2009;**4**:1–6. doi:10.1371/journal.pone.0007484

8 Cecil E, Bottle A, Sharland M, *et al.* Impact of UK primary care policy reforms on shortstay unplanned hospital admissions for children with primary care–sensitive conditions. *Annals of Family Medicine* 2015;**13**:214–20. doi:10.1370/afm.1786

9 Gill PJ, Goldacre MJ, Mant D, *et al.* Increase in emergency admissions to hospital for children aged under 15 in England, 1999-2010: National database analysis. *Archives of Disease in Childhood* 2013;**98**:328–34. doi:10.1136/archdischild-2012-302383

10 Wyatt S, Mohammed MA, Fisher E, *et al.* Impact of the SARS-CoV-2 pandemic and associated lockdown measures on attendances at emergency departments in English hospitals: A retrospective database study. *The Lancet Regional Health - Europe* 2021;**2**:100034. doi:10.1016/j.lanepe.2021.100034

11 Hartshorn S, O'Sullivan R, Maconochie IK, *et al.* Establishing the research priorities of paediatric emergency medicine clinicians in the UK and Ireland. *Emergency Medicine Journal* 2015;**32**:864–8. doi:10.1136/emermed-2014-204484

12 Miller SZ, Rincón H, Kuppermann N, *et al.* Revisiting the Emergency Medicine Services for Children Research Agenda: Priorities for Multicenter Research in Pediatric Emergency Care. *Academic Emergency Medicine* 2008;**15**:377–83. doi:https://doi.org/10.1111/j.1553-2712.2008.00072.x

13 Bialy L, Plint AC, Freedman SB, *et al.* Pediatric Emergency Research Canada (PERC): Patient/Family-informed Research Priorities for Pediatric Emergency Medicine. *Academic Emergency Medicine* 2018;**25**:1365–74. doi:https://doi.org/10.1111/acem.13493

14 Jones CHD, Howick J, Roberts NW, *et al.* Primary care clinicians' attitudes towards point-of-care blood testing: A systematic review of qualitative studies. *BMC Family Practice* 2013;**14**. doi:10.1186/1471-2296-14-117

15 Schito M, Peter T, Cavanaugh J, *et al.* Opportunities and Challenges for Cost-Efficient Implementation of New Point-of-Care Diagnostics for HIV and Tuberculosis. *J Infect Dis* 2012;**205 Suppl 2**:S169-80. doi:10.1093/infdis/jis044

16 Fujita-Rohwerder N, Beckmann L, Zens Y, *et al.* Diagnostic accuracy of rapid point-of-care tests for diagnosis of current SARS-CoV-2 infections in children: a systematic review and meta-analysis. *BMJ Evidence-Based Medicine* 2022;:bmjebm-2021-111828. doi:10.1136/bmjebm-2021-111828

For peer review only - http://bmjopen.bmj.com/site/about/guidelines.xhtml

17 Li E, Dewez JE, Luu Q, *et al.* Role of point-of-care tests in the management of febrile children: a qualitative study of hospital-based doctors and nurses in England. *BMJ Open* 2021;**11**:e044510. doi:10.1136/bmjopen-2020-044510

18 Pandey M, Lyttle MD, Cathie K, *et al.* Point-of-care testing in Paediatric settings in the UK and Ireland: a cross-sectional study. *BMC Emergency Medicine* 2022;**22**. doi:10.1186/s12873-021-00556-7

19 Rasti R, Brännström J, Mårtensson A, *et al.* Point-of-care testing in a high-income country paediatric emergency department: A qualitative study in Sweden. *BMJ Open* 2021;**11**. doi:10.1136/bmjopen-2021-054234

20 Silverman D. *Doing qualitative research : a practical handbook* . 3rd ed. London ; : SAGE 2010.

21 Patton MQuinn. *How to use qualitative methods in evaluation* . Newbury Park, Calif: : Sage Publications 1987.

22 Strauss A, Corbin J. *Basics of Qualitative Research: Techniques and Procedures for Developing Grounded Theory*. 2nd ed. Thousand Oaks, CA: Sage 1990.

23 Flick U. The SAGE Handbook of Qualitative Data Analysis. 2014. doi:10.4135/9781446282243

24 Dixon S, Glogowska M, Garland S, *et al.* Clinician perspectives on having point of care tests made available to them during out of hours home visiting. *BMC Family Practice* 2021;**22**:246. doi:10.1186/s12875-021-01571-0

25 Butler J, de Cassan S, Turner P, *et al.* Attitudes to physical healthcare in severe mental illness; a patient and mental health clinician qualitative interview study. *BMC Family Practice* 2020;**21**:243. doi:10.1186/s12875-020-01316-5

26 Durand MA, Chantler T, editors. *Principles of social research* . Second edi. Maidenhead, England: : Open University Press 2014.

27 NHS. Integrating Care: 2021. doi:10.4324/9781315735320

28 Anderson M, Pitchforth E, Asaria M, *et al.* LSE–Lancet Commission on the future of the NHS: re-laying the foundations for an equitable and efficient health and care service after COVID-19. *The Lancet* 2021;**397**:1915–78. doi:https://doi.org/10.1016/S0140-6736(21)00232-4

29 Panicker L. Nurses' Perceptions of Parent Empowerment in Chronic Illness. *Contemp Nurse* 2013;**45**:210–9. doi:10.5172/conu.2013.45.2.210

30 Van Den Bruel A, Jones C, Thompson M, *et al.* C-reactive protein point-of-care testing in acutely ill children: A mixed methods study in primary care. *Archives of Disease in Childhood* 2016;**101**:382–6. doi:10.1136/archdischild-2015-309228

For peer review only

Table 1 Complete participant characteristics

Participant	Job role	Time in that role /years (mo= months)	Gender	Level 1= primary 2= secondary 3= tertiary	Setting Rural 0 Urban 1	Recruitment 1= PERUKI 2=Website 3=Direct 4=Snowball 5=conference
Clinicians						
01	Consultant paediatric and neonatal surgeon<br>BAPS	5<br>2	M	3<br>n/a	1	3
02	Consultant paediatrician	2	M	2	0	1
03	GP	1 mo	F	1	1	4
04	Specialist asthma nurse	26	F	1	1	4
05	Macmillan GP<br>CCG clinical lead for children and young people	12<br>5 mo	F	1<br>n/a	1	3
06	Consultant in paediatric and adult emergency medicine	14	F	2	1	1
07	Specialist paediatric trainee	5	F	2	1	4
08	Foundation Year 1 Doctor (junior doctor in their first year of practice)	2 mo	M	2	1	4
09	GP<br>CCG clinical lead for cancer, children & maternity	20<br>8	M	1<br>n/a	1	2
10	GP	4	F	1	1	5
11	Consultant children's orthopaedic surgeon	4	M	3	1	3
12	Primary care advanced nurse practitioner	35	F	1	0	4
13	Consultant community paediatrician	21	M	2	Mixture	3
14	Consultant community psychiatrist of children and adolescents	3.5	F	2	Mixture	3
15	Senior staff nurse children's emergency department	4	F	3	1	3
16	Urgent care GP	18	M	1	1	2
17	Primary care advanced nurse practitioner	23	F	1	1	3
Stakeholders						
01	Meningitis Research Foundation	2	F	n/a	n/a	3

For peer review only - http://bmjopen.bmj.com/site/about/guidelines.xhtml

Participant	Job role	Time in that role /years (mo= months)	Gender	Level 1= primary 2= secondary 3= tertiary	Setting Rural 0 Urban 1	Recruitment 1= PERUKI 2=Website 3=Direct 4=Snowball 5=conference
02	Little Miracles	10	F	n/a	n/a	3
03	Asthma UK<br>GP	3<br>15	M	n/a<br>1	Mixture	3
04	HappyR health	1	M	n/a	n/a	3
05	CCG clinical lead for children, young people & maternity<br>GP	20<br>7	F	n/a<br>1	1	3

BAPS, British Association of Paediatric Surgeons, GP General Practitioner, CCG Clinical Commissioning Group, PERUKI Paediatric Emergency Research in the UK and Ireland. N/A Not Applicable. Participants highlighted in grey are both clinicians and stakeholders.

Table 2 Main themes and subthemes

Theme 1: Potential benefits of POC tests and technologies
1a: POC tests facilitate early decision-making
1b: Home-based POC tests are convenient
1c: POC tests are empowering for children and their families
Theme 2: Areas for improvement for POC tests and technologies
2a: POC tests should be more widely available
2b: End-users should find POC tests quick and easy to use
2c: POC tests should be agreeable and engaging for children
2d: POC tests should make a difference to clinical management

For peer review only - http://bmjopen.bmj.com/site/about/guidelines.xhtml

Table 3 Additional participant quotes listed by theme and sub-theme

Theme	Sub-theme	Test/technology	Quote	Participant
**1: Potential benefits of POC tests and technologies**	1a POC tests facilitate early decision-making	Spirometry, FeNO	*"Tests, such as, spirometry and FeNO are good objective measures which we can use at the bedside to help decide whether… somebody has or doesn't have asthma… a lot of patients get under diagnosed… that means they're getting chronic symptoms and inflammation and ongoing damage within the airways… which can cause… disability from stopping them doing normal things in their life; it can put them at risk of life-threatening asthma attacks and it can cause chronic inflammation of the lungs causing long-term damage."*	Stakeholder#3
	1b: Home-based POC tests are convenient	Remote observations	*"from a patient perspective and a practice perspective… seeing as much as we can remotely is… much better. Nobody in their right mind wants to bring a sick child out and sit in a doctor's surgery waiting for a doctor or practitioner to be running late [when] the kid's not well"*	Clinician#17
**2: Areas for improvement for POC tests and technologies**	2b: End-users should find POC tests quick and easy to use	Urinalysis	*"we had an example of a [teenage] girl… with fairly non-specific symptoms… Had not been able to produce the urine, said they would do it later, that didn't happen… the diagnosis was made about perhaps a week later [of] diabetes"*	Clinician#12
		Smart inhaler	*"there is one device that clips to one specific inhaler… it measures the sound of the inhalation so you can gauge whether or not… that dose has been taken properly… currently it's only being used in research, but the potential is there"*	Clinician#4
		Monitoring of exhaled gases	*" before long there will be the technology that when you talk into your mobile phone it will be able to monitor your asthma… a combined exhaled carbon monoxide and nitric oxide monitor"*	Clinician#4

POC point-of-care, FeNO Fractional exhaled Nitric Oxide

For peer review only

Table 4 Unmet needs: Ideas for application of new tests or technologies that have not already been mentioned in Table 3

Test/technology/pathway	Quote	Participant
**Acute serious illness**		
Predicting severity of bronchiolitis	*"it's really difficult to tell which, which babies are going to have a mild broncholitic course and just settle down quite quickly and those that are going to progress and need additional respiratory support, so… whether there's a breath-activated test… that tells you… [that] would be incredible"*	Clinician#2
Remote observations using smartphone cameras and apps	*"we… are wary of sepsis for example…. in children who are poorly with acute illnesses we… spend quite a lot of time gaining information about those particular sepsis markers so I will be checking their oxygen levels, I'll be measuring their respiratory rate. I'll be checking their pulse. I'll be checking their blood pressure if that's appropriate. We'll be checking their temperature, their capillary refill time… if a patient could do that [at home] so there is an App which can [quickly and non-invasively] assess these [sepsis] markers… that would be hugely helpful… in making a decision safely…and may mean that less patients need to be assessed face to face or in hospital… it would save us a lot of time and would provide a lot of assistance"*	Clinician#15
Poisons and seizures	*"you can send the blood test off and get paracetamol salicylate levels; that's fairly standard… It would be helpful to get those results earlier [with] other drugs… for your older teenager who comes in unconscious and you're wondering what they might have taken…. children with epilepsy… are they taking the right dose of sodium valproate?... if you could find that out quickly then would, that would change our management… when they're coming in having a seizure"*	Clinician#6
Appendicitis	*"if you had a child who was suspected to have appendicitis clinically, but you wanted to be more certain, then you would have access to… a bedside ultrasound… and prove definitively whether they did or did not… 1) it could provide better selection of children who needed to have treatment for their appendicitis; and 2)… it could give reassurance to those who didn't have appendicitis so they could be sent home"*	Clinician#1
Ovarian torsion	*"ultrasound is used for ovarian torsion… [that] could be done at the bedside"*	Clinician#1
Fracture	*"avoiding X-rays, doing near patient ultrasound to diagnose your fracture or whatever it is. … some of this stuff can really help with minors, reducing radiation exposure of children and, and speeding up the process"*	Clinician#6
**Distinguishing bacterial and viral infection**		
Diagnosing bacterial meningitis	*"you could distinguish viral meningitis and bacterial meningitis to high sensitivity and specificity with this 2 RNA transcript signature"*	Stakeholder#1
	"I have read about the rapid DNA test for Neisseria meningitis… and that will be very useful in the context of a child presenting with non-blanching rash and fever… I tend to over treat these kind of children or to admit for observations waiting for… blood tests to come back"	Clinician#7
**Diagnosing and monitoring chronic disease**		
Assessing pain or stress in children unable to communicate	*"kids with ASD … you could monitor where [and] when their heart rate goes up and when there's more signs of stress, even if they don't realise that they're getting stressed at these times… some objective monitoring could be helpful for those kids because they're not very aware of their own emotions...you can [then] plan an intervention accordingly".*	Clinician#14
Diagnosing genetic diseases	*"we're talking of whole genetic sequencing coming along very, very quickly now…getting the results by the bedside"*	Clinician#13

RNA, ribonucleic acid; ASD, autistic spectrum disorder

For peer review only

Supplementary Materials: Interview Topic Guide

Version 1.1 09.01.2020

Thank you for participating in our study. Any questions about the information booklet?

Background
Our aim is to understand when, where, how and why point-of-care tests and technologies in ambulatory paediatrics could be useful. By ambulatory settings, we mean primary care, emergency departments and out-of-hours services. By point-of-care tests, we mean any diagnostic technology to include tests on bodily fluids, imaging, wearables, digital technology, and smart phone apps.

Consent form

Interview
Recording now….

All:
- What is your job description?
- How many years' experience do you have in that role?
- Age
- What area of the country do you work in?
- Would you say that your work setting is:

Rural Urban
Primary Care Secondary Care Tertiary Care
(Stakeholder)

All:
What do you think about bedside **TESTS** in children in ambulatory care settings?

What experience do you have of these?

What current point-of-care tests are currently useful in clinical practice (stakeholders: have you heard of existing tests being useful)?
Probe e.g. urine dip, blood glucose check

How have these tests been helpful in your clinical practice (stakeholders: have you heard of existing tests being useful)? Please give an example. How did it change what you did?

Do you have tests available to you that aren't useful (stakeholders: are there tests that aren't useful)? Please give an example.
Probe: BM when not confident to do a finger prick, too time consuming
Reasons why not

Have **TECHNOLOGIES** ever been helpful in your clinical practice? Example if yes.
Probe: apps for fitting, temperature monitors

Clinicians:

For peer review only

What training have you had in the use of POC testing/technology?

Which clinical pathways might benefit from a new test/technology?

Can you think of a recent specific situation in which it would have been useful to have a novel point-of-care test or technology?

How would that be useful? How would it change what you would do, or the patient outcome?

Probe:

for:

- *Decision making e.g.*
  - *Treatment given e.g. Antibiotic prescribing*
  - *Predicting severity of illness*
  - *Referral to secondary care*
  - *Admission to hospital*
  - *Self-management*
  - *Hospital management- investigations, treatments, referral, surgical, length of stay*
- *Waiting times*
- *Explanation of treatment plan*

Where would you see them fitting in the pathway of patient flow?

What would be key characteristics of a successful paediatric diagnostic?

Probe: "facilitators" in terms of….

- *time taken to perform the test*
- *time taken to obtain the result*
- *cost*
- *route of sampling*
- *amount of tests able to perform simultaneously (e.g. panel)*
- *Acceptability: Who would perform? (Ease of use), Novelty, trust*
- *Sensitivity and specificity (uncertainty)*

What impact might POC tests/technology have on *parents*? What would they need to know?

What *disadvantages* might POC tests/technology in paediatrics in ambulatory settings have?

Probe: barriers to use

Are you aware of any new promising diagnostics in development coming out in your work place?

Do you have any other thoughts or ideas?

Any questions for me?

Is there anyone else that you can think of who might want to contribute**?**

Admin

I will send Amazon voucher by email

Would you like a CPD certificate to acknowledge your contribution?

For peer review only

Standards for Reporting Qualitative Research (SRQR)*

http://www.equator-network.org/reporting-guidelines/srqr/

Page/line no(s).

Title and abstract

**Title** - Concise description of the nature and topic of the study Identifying the study as qualitative or indicating the approach (e.g., ethnography, grounded theory) or data collection methods (e.g., interview, focus group) is recommended	p1
**Abstract** - Summary of key elements of the study using the abstract format of the intended publication; typically includes background, purpose, methods, results, and conclusions	p2

Introduction

**Problem formulation** - Description and significance of the problem/phenomenon studied; review of relevant theory and empirical work; problem statement	3-4
**Purpose or research question** - Purpose of the study and specific objectives or questions	4

Methods

**Qualitative approach and research paradigm** - Qualitative approach (e.g., ethnography, grounded theory, case study, phenomenology, narrative research) and guiding theory if appropriate; identifying the research paradigm (e.g., postpositivist, constructivist/ interpretivist) is also recommended; rationale**	4-5
**Researcher characteristics and reflexivity** - Researchers' characteristics that may influence the research, including personal attributes, qualifications/experience, relationship with participants, assumptions, and/or presuppositions; potential or actual interaction between researchers' characteristics and the research questions, approach, methods, results, and/or transferability	6
**Context** - Setting/site and salient contextual factors; rationale**	4-5
**Sampling strategy** - How and why research participants, documents, or events were selected; criteria for deciding when no further sampling was necessary (e.g., sampling saturation); rationale**	5
**Ethical issues pertaining to human subjects** - Documentation of approval by an appropriate ethics review board and participant consent, or explanation for lack thereof; other confidentiality and data security issues	6
**Data collection methods** - Types of data collected; details of data collection procedures including (as appropriate) start and stop dates of data collection and analysis, iterative process, triangulation of sources/methods, and modification of procedures in response to evolving study findings; rationale**	5

For peer review only - http://bmjopen.bmj.com/site/about/guidelines.xhtml

**Data collection instruments and technologies** - Description of instruments (e.g., interview guides, questionnaires) and devices (e.g., audio recorders) used for data collection; if/how the instrument(s) changed over the course of the study	5
**Units of study** - Number and relevant characteristics of participants, documents, or events included in the study; level of participation (could be reported in results)	6, Table 1
**Data processing** - Methods for processing data prior to and during analysis, including transcription, data entry, data management and security, verification of data integrity, data coding, and anonymization/de-identification of excerpts	5
**Data analysis** - Process by which inferences, themes, etc., were identified and developed, including the researchers involved in data analysis; usually references a specific paradigm or approach; rationale**	6
**Techniques to enhance trustworthiness** - Techniques to enhance trustworthiness and credibility of data analysis (e.g., member checking, audit trail, triangulation); rationale**	6

Results/findings

**Synthesis and interpretation** - Main findings (e.g., interpretations, inferences, and themes); might include development of a theory or model, or integration with prior research or theory	6-12
**Links to empirical data** - Evidence (e.g., quotes, field notes, text excerpts, photographs) to substantiate analytic findings	6-10

Discussion

**Integration with prior work, implications, transferability, and contribution(s) to the field** - Short summary of main findings; explanation of how findings and conclusions connect to, support, elaborate on, or challenge conclusions of earlier scholarship; discussion of scope of application/generalizability; identification of unique contribution(s) to scholarship in a discipline or field	10-12
**Limitations** - Trustworthiness and limitations of findings	11

Other

**Conflicts of interest** - Potential sources of influence or perceived influence on study conduct and conclusions; how these were managed	13
**Funding** - Sources of funding and other support; role of funders in data collection, interpretation, and reporting	13

*The authors created the SRQR by searching the literature to identify guidelines, reporting standards, and critical appraisal criteria for qualitative research; reviewing the reference lists of retrieved sources; and contacting experts to gain feedback. The SRQR aims to improve the transparency of all aspects of qualitative research by providing clear standards for reporting qualitative research.

For peer review only

For peer review only - http://bmjopen.bmj.com/site/about/guidelines.xhtml

**The rationale should briefly discuss the justification for choosing that theory, approach, method, or technique rather than other options available, the assumptions and limitations implicit in those choices, and how those choices influence study conclusions and transferability. As appropriate, the rationale for several items might be discussed together.

Reference:

O'Brien BC, Harris IB, Beckman TJ, Reed DA, Cook DA. **Standards for reporting qualitative research: a synthesis of recommendations.** *Academic Medicine*, Vol. 89, No. 9 / Sept 2014 DOI: 10.1097/ACM.0000000000000388

For peer review only
